# MV-ComBat and MV-CovBat: Multivariate Frameworks for Joint Harmonization of Multi-Metric Neuroimaging Data

**DOI:** 10.64898/2026.02.05.704069

**Published:** 2026-02-09

**Authors:** Zheng Ren, Patrick Sadil, Martin A. Lindquist

**Affiliations:** Department of Biostatistics, Johns Hopkins Bloomberg School of Public Health, 615 N. Wolfe Street, Baltimore, Maryland 21205, USA

**Keywords:** neuroimaging, harmonization, ComBat, multivariate

## Abstract

Aggregating neuroimaging data across sites and studies is increasingly common, yet site- and scanner-related batch effects can obscure meaningful biological variation and introduce spurious associations. Although ComBat and its extensions are widely used, they are primarily designed for single-metric (univariate) harmonization. In practice, neuroimaging studies often involve multiple biologically coupled metrics (e.g., cortical thickness, surface area, and gray-matter volume) measured across multiple features (e.g., regional values), with shared covariance structure both within and across metrics. Applying univariate ComBat independently to each metric ignores these dependencies and can leave residual batch effects in cross-metric covariance. Using data from the NIH Acute to Chronic Pain Signatures (A2CPS) program, we show that batch effects occur not only in means and variances but also in covariance across cortical regions and metrics—relationships that univariate ComBat does not fully remove. We propose MV-ComBat, a multivariate extension of ComBat that jointly harmonizes multiple metrics by borrowing strength across them. Both empirical Bayes (EB) and Bayesian Markov Chain Monte Carlo (MCMC) implementations of MV-ComBat effectively reduce batch effects. In our experiments, EB is more robust to measurement error, whereas MCMC more accurately recovers cross-metric correlations when priors are well specified. Recognizing that batch effects can also affect feature-level covariance, CovBat was recently introduced as an extension of ComBat that harmonizes both first- and second-order moments across sites. We extend CovBat to the multivariate framework as MV-CovBat, which performs a second-stage latent-space harmonization to address covariance-related batch effects across features and metrics. Simulations confirm that MV-ComBat improves correlation recovery and biological signal preservation relative to univariate ComBat, particularly for moderate-to-strong effects, and that MV-CovBat further improves separation of true biological variation from batch effects when independence assumptions are violated. Together, these methods provide a flexible and unified framework for harmonizing complex, multi-metric neuroimaging data in large-scale, multi-site studies.

## 1 Introduction

Pooling neuroimaging data across sites and scanners is increasingly necessary to achieve adequate sample sizes and improve generalizability. There is also growing interest in integrating multi-domain data to enable comprehensive biomarker discovery. One initiative exemplifying this trend is the National Institutes of Health (NIH)-funded *Acute to Chronic Pain Signatures (A2CPS)* program, which aims to identify and validate biomarkers of pain chronification by jointly analyzing genomic, proteomic, metabolomic, lipidomic, neuroimaging, psychophysical, psychological, and behavioral measures (Sluka et al. 2023). Such large-scale, multi-site, and multi-modal efforts highlight both the promise of integrative analyses and the challenges posed by technical and site-specific variability. Imaging data are particularly susceptible, because heterogeneous acquisition protocols and scanner parameters can introduce site- and scanner-related differences, commonly referred to as batch effects. These effects are not biologically meaningful, yet they can confound associations of interest, reduce reproducibility, and limit interpretability. Effective harmonization methods are therefore essential to remove non-biological variability while preserving biologically meaningful signal.

A wide range of statistical harmonization approaches have been developed, among which ComBat has become one of the most widely adopted. Originally proposed for genomic data to correct batch effects in high-dimensional expression arrays (Johnson, Li, and Rabinovic 2006), it has since been successfully applied to diverse neuroimaging modalities, including diffusion tensor imaging (DTI) (Fortin, Parker, et al. 2017), cortical thickness (Fortin, Cullen, et al. 2018), functional connectivity (Yu et al. 2018), and radiomic features derived from positron emission tomography (PET) (Orlhac et al. 2018). ComBat models and removes site- or scanner-related shifts in mean and variance using an empirical Bayes approach, thereby reducing unwanted variability while preserving meaningful biological associations. Several extensions have been proposed to accommodate more complex data structures, including ComBat-GAM (Pomponio et al. 2020) for nonlinear covariate effects, LongComBat (Beer et al. 2020) for longitudinal data, and CovBat (Chen, Beer, et al. 2021) for harmonizing feature covariance across batches to enhance downstream machine learning analyses. More recently, a fully Bayesian version of ComBat has been introduced (Reynolds et al. 2023), which may better preserve biological variation while achieving more accurate harmonization.

Despite these important advances, traditional ComBat and its variants are primarily designed for single-metric (i.e., univariate) data. In practice, however, neuroimaging analyses often involve multiple biologically coupled metrics derived from the same imaging modality (e.g., cortical thickness, surface area, and gray-matter volume from T1-weighted MRI) or across modalities (e.g., T1/T2 ratios and diffusion-derived metrics). Each metric comprises multiple features (e.g., cortical regions) that share structured covariance reflecting underlying biological processes. For example, across the adult lifespan, there is a dynamic relationship between cortical thickness and surface area (Storsve et al. 2014), and morphometric similarity network analyses suggest macroscale co-alteration patterns (Lu et al. 2024). Wu et al. 2021 analyzed several cortical structural measures across brain regions in relation to Alzheimer’s disease risk, illustrating multimetric analysis within a single framework, in which results can be sensitive to systematic variation across metrics. More recently, Sadikov et al. 2025 demonstrated that diffusion MRI yields dozens of reliably measurable cortical microstructural metrics that exhibit substantial shared variance, which can be summarized by a smaller number of latent factors. Applying univariate ComBat independently to each metric ignores these dependencies, leaving residual batch effects in covariance structure and potentially attenuating true biological variation.

To address this limitation, we propose MV-ComBat, a multivariate extension of the ComBat framework that jointly harmonizes multiple metrics by borrowing strength across correlated features and metrics. By explicitly modeling shared covariance structure, MV-ComBat more effectively removes batch effects in both means and covariances while preserving biologically meaningful relationships across metrics and features. This framework is particularly advantageous for studies investigating joint associations across multiple feature sets or metrics, but is also applicable to metric-specific analyses, where harmonization benefits from information sharing across correlated metrics.

Recognizing that batch effects can also be present in feature-level covariance, CovBat was introduced as an extension of ComBat that harmonizes both first- and second-order moments across sites within each metric. Building on this idea, we extend CovBat to the multivariate setting with multiple metrics and introduce MV-CovBat, which removes batch effects in covariance both within and across metrics. Compared with MV-ComBat, this method adds a second-stage harmonization step in a latent covariance space to mitigate residual batch effects that may persist when independence assumptions are violated. By explicitly addressing covariance structure, MV-CovBat further improves harmonization of cross-feature and cross-metric relationships while preserving biologically meaningful variability.

We demonstrate the utility of these methods using data from the A2CPS project, showing that MV-ComBat more effectively reduces batch effects than univariate ComBat, both within and across metrics. The CovBat framework generally performs better at removing global batch effects across features, while MV-CovBat further leverages information across metrics to more effectively disentangle batch effects from biological variation, achieving the best overall performance, particularly for noisy metrics with unknown confounding effects. Simulation studies further confirm these improvements, with superior preservation of biological signals and more accurate recovery of true correlation structure. Together, MV-ComBat and MV-CovBat provide a flexible and unified framework for harmonizing complex, multi-metric neuroimaging data in large-scale, multi-site studies.

## 2 Method

### 2.1 A2CPS dataset

The data analyzed in this study consist of baseline scans (release 1.0.0) from the Acute to Chronic Pain Signatures (A2CPS) program, funded by the National Institutes of Health (NIH) Common Fund. A2CPS aims to identify biomarkers and biosignatures across multiple data types that, individually or jointly, predict susceptibility or resilience to developing chronic pain after an acute pain event (Sluka et al. 2023). Informed consent was obtained from all participants. Detailed inclusion criteria and imaging acquisition protocols are described in Berardi et al. 2022.

A2CPS imaging data are collected across multiple sites using scanners from General Electric (GE), Siemens, and Philips, with protocols adapted from the Adolescent Brain Cognitive Development (ABCD) study; see Sadil et al. 2024 for more details. In this work, we focus on T1-weighted structural MRI data that provide multiple imaging-derived metrics across cortical regions. Each A2CPS T1-weighted scan received a quality rating on a three-point scale (“green”, “yellow”, “red”), where “yellow” and “red” indicate potential quality concerns. Accordingly, only scans rated “green” were included in this study.

Preprocessing was performed using *fMRIPrep* (Esteban et al. 2018). The T1-weighted (T1w) images were corrected for intensity non-uniformity (INU) using N4BiasFieldCorrection (Tustison et al. 2010) and served as the anatomical reference throughout the workflow. Skull stripping was performed using a Nipype implementation of the ANTs brain extraction workflow (antsBrainExtraction.sh), with the OASIS30ANTs template as the target (Avants et al. 2011). Cortical surface reconstruction was carried out using FreeSurfer (recon-all; Fischl 2012).

Harmonization performance of existing methods may decline as the number of features increases, likely due to the increasingly complex covariance structures across features and potential confounding with biological variables. Prior studies have shown that, for covariance-based harmonization methods such as CovBat, site effects may become more detectable after harmonization in settings with relatively small sample sizes and high-dimensional feature spaces, potentially due to poor covariance estimation (Chen, Beer, et al. 2021). To rigorously evaluate harmonization performance across methods, we therefore consider a deliberately challenging scenario. Specifically, we focus on the Destrieux cortical parcellation, which includes 78 regions of interest (ROIs), each characterized by eight different cortical metrics: surface area (SurfArea), gray-matter volume (GrayVol), mean thickness (ThickAvg), thickness standard deviation (ThickStd), mean curvature (MeanCurv), Gaussian curvature (GausCurv), folding index (FoldInd), and curvature index (CurvInd). These metrics include quantities with known mathematical and biological relationships, as well as differing statistical scales, thereby providing a stringent test of harmonization methods in high-dimensional and correlated settings.

The analytic sample included 473 participants (34% male; mean age = 63 years; mean self-reported pre-surgical pain duration = 53 months) from six sites and three scanner manufacturers: Wayne State (WS; Siemens, *n* = 29), University of Illinois Chicago (UI; GE, *n* = 130), University of Michigan (UM; GE, *n* = 97), Spectrum Health (SH; Siemens, *n* = 22), University of Chicago (UC; Philips, *n* = 82) and Endeavor Health (NS; Siemens, *n* = 113). Site and scanner differences represent a major source of potential batch effects. Effective harmonization is essential to mitigate these effects while preserving biologically relevant variation, thereby improving the reliability of downstream analyses and predictive modeling.

### 2.2 Review of ComBat-based methods

We first review commonly used ComBat-based methods for harmonizing neuroimaging data. The original ComBat (Johnson, Li, and Rabinovic 2006) and its extensions aim to remove batch effects in feature-wise mean and variance within an empirical Bayes (EB) framework. Here, fixed covariate effects are estimated and preserved via regression, while batch-specific location and scale parameters are estimated via EB posterior means, with prior hyperparameters obtained using method-of-moments (MoM) estimation across features.

The methods vary primarily in how they model the underlying biological signal and data structure. *ComBat* assumes cross-sectional data with approximately linear covariate effects and harmonizes each feature independently. *LongComBat* extends this framework to longitudinal data by incorporating subject-specific random effects, *ComBat–GAM* uses smooth functions to capture nonlinear covariate effects, and *CovBat* further adjusts the covariance structure across features to reduce batch-related differences in multivariate correlations.

#### Original ComBat.

The original *ComBat* is designed for cross-sectional data with approximately linear covariate effects. Let $y_{ijv}$ denote the value of feature v for subject j in batch i, and let $X_{ij}$ be a vector of covariates. *ComBat* models each feature as

$$y_{ijv}=\alpha_{v}+X_{ij}^{⊤}\beta_{v}+\gamma_{iv}+\delta_{iv}\epsilon_{ijv},\;\epsilon_{ijv}\overset{\;\text{i.i.d.}\;}{∼}𝒩\left(0,\sigma_{v}^{2}\right)$$

where $\alpha_{v}$ is a feature-specific intercept, $\beta_{v}$ are fixed-effect coefficients, and $\gamma_{iv}$ and $\delta_{iv}$ represent batch-specific location (mean) and scale (variance) effects. For each feature v, least-squares estimates ${\overset{ˆ}{\alpha }}_{v}$ and ${\overset{ˆ}{\beta }}_{v}$ are obtained by regression. Empirical Bayes priors are then placed on the batch effects across features, with hyperparameters estimated by MoM. Posterior means $\gamma_{iv}^{*}$ and $\delta_{iv}^{*}$ are used to adjust the data while preserving the fixed effects, yielding the harmonized values:

$$y_{ijv}^{ComBat}=\frac{y_{ijv}\;-\;{\overset{ˆ}{\alpha }}_{v}\;-\;X_{ij}^{⊤}{\overset{ˆ}{\beta }}_{v}\;-\;\gamma_{iv}^{*}}{\delta_{iv}^{*}}+{\overset{ˆ}{\alpha }}_{v}+X_{ij}^{T}{\overset{ˆ}{\beta }}_{v}.$$

#### LongComBat.

*LongComBat* extends the original ComBat framework to longitudinal data, where participants have repeated measurements over time. For feature v of subject j in batch i at time t, the model is

$$y_{ijv}(t)=\alpha_{v}+X_{ij}(t{)}^{⊤}\beta_{v}+\eta_{jv}+\gamma_{iv}+\delta_{iv}\varepsilon_{ijv}\left(t\right),\;\varepsilon_{ijv}\left(t\right)\overset{\;\text{i.i.d.}\;}{∼}𝒩\left(0,\sigma_{v}^{2}\right),$$

where $\eta_{jv}$ is a subject-specific random effect, $\gamma_{iv}$ and $\delta_{iv}$ are batch-specific location and scale effects, and $X_{ij}(t)$ contains biological covariates to be preserved. Fixed and random effects are first estimated using restricted maximum likelihood (REML). Similar to *ComBat*, batch effects are then estimated in an EB framework, with hyperparameters obtained via MoM, and posterior means of the batch effects subsequently used for adjustment. The harmonized values are

$$y_{ijv}^{\mathrm{LongComBat}}(t)=\frac{y_{ijv}(t)\;-\;{\overset{ˆ}{\alpha }}_{v}\;-\;X_{ij}(t{)}^{⊤}{\overset{ˆ}{\beta }}_{v}\;-\;\gamma_{iv}^{*}\;-\;{\overset{ˆ}{\eta }}_{jv}}{\delta_{iv}^{*}}+{\overset{ˆ}{\alpha }}_{v}+X_{ij}(t{)}^{T}{\overset{ˆ}{\beta }}_{v}+{\overset{ˆ}{\eta }}_{jv}.$$

This formulation enables harmonization of repeated measures while accounting for within-subject correlations and between-site variability.

#### ComBat-GAM.

For cross-sectional data with nonlinear covariate effects, *ComBat–GAM* replaces the linear term with a smooth function:

$$y_{ijv}=f_{v}\left(X_{ij},Z_{ij}\right)+\gamma_{iv}+\delta_{iv}\varepsilon_{ijv},\;f_{v}\left(X_{ij},Z_{ij}\right)=\alpha_{v}+f_{v}\left(X_{ij}\right)+Z_{ij}^{⊤}\beta_{v},$$

where $f_{v}(\cdot )$ is a spline-based smooth function of selected covariates for feature v, $X_{ij}$ contains nonlinear covariates, and $Z_{ij}$ contains linear covariates. Estimation follows the standard *ComBat* EB pipeline, with ${\overset{ˆ}{f}}_{v}(\cdot )$ obtained via generalized additive models (GAM). Posterior means $\left(\gamma_{iv}^{*},\delta_{iv}^{*}\right)$ are then used for harmonization. The harmonized values are

$$y_{ijv}^{ComBat-GAM}=\frac{y_{ijv}\;-\;{\overset{ˆ}{f}}_{v}\left(X_{ij},Z_{ij}\right)\;-\;\gamma_{iv}^{*}}{\delta_{iv}^{*}}+{\overset{ˆ}{f}}_{v}\left(X_{ij},Z_{ij}\right)$$

This formulation captures nonlinear biological effects while removing site-related shifts in mean and variance.

#### CovBat.

The three methods above primarily focus on batch effects in feature-wise means and scales. However, they do not correct batch effects in cross-feature covariance, which can bias association estimates and degrade downstream machine learning performance. *CovBat* extends the *ComBat* framework to harmonize covariance through a two-stage procedure.

##### Stage 1 (Feature-wise ComBat).

Standard ComBat is first applied to remove batch-specific mean and variance effects from each feature's marginal distributions, providing residuals

$$e_{ijv}^{ComBat}=\frac{y_{ijv}\;-\;\overset{ˆ}{g}\left(X_{ij}\right)\;-\;\gamma_{iv}^{*}}{\delta_{iv}^{*}},$$

so that, for each batch i, the residual vector $e_{ij}^{\text{ComBat}}={\left(e_{ij1}^{\text{ComBat}},\ldots ,e_{ijp}^{\text{ComBat}}\right)}^{⊤}$ has mean zero and batch-specific covariance $\Sigma_{i}$. Here, p is the total number of features, and $\overset{ˆ}{g}(\cdot )$ denotes the estimated contribution of the preserved biological effects.

##### Stage 2 (PCA-domain covariance harmonization).

Principal component analysis (PCA) is performed on the pooled residual covariance matrix to obtain eigenvectors $\left\{{\overset{ˆ}{\phi }}_{1},\ldots ,{\overset{ˆ}{\phi }}_{p}\right\}$ and principal component (PC) scores $\xi_{ijk}={\overset{ˆ}{\phi }}_{k}^{⊤}e_{ij}^{\text{ComBat}}$. The first K score coordinates are harmonized across batches to produce $\xi_{ijk}^{\text{CovBat}}$, and the harmonized residuals are reconstructed as:

$$e_{ij}^{\text{CovBat}\;}=\sum_{k=1}^{K}\xi_{ijk}^{\text{CovBat}\;}{\overset{ˆ}{\phi }}_{k}+\sum_{k=K+1}^{p}\xi_{ijk}{\overset{ˆ}{\phi }}_{k}.$$

Here K controls the fraction of total variance whose covariance structure is harmonized. Finally, the preserved fixed effects are added back to obtain CovBat-adjusted observations:

$$y_{ijv}^{\text{CovBat}\;}=e_{ijv}^{\text{CovBat}\;}+\overset{ˆ}{g}\left(X_{ij}\right).$$

This two-stage approach harmonizes both marginal distributions and the cross-feature covariance across batches, improving consistency for downstream multivariate analyses.

### 2.3 Multivariate ComBat

The four ComBat-based methods described above have demonstrated promising harmonization performance in single-metric scenarios, which we refer to collectively as the univariate ComBat framework. However, neuroimaging analyses often involve multiple correlated metrics (e.g., cortical thickness, surface area, curvature) that share biologically meaningful covariance both within and across cortical regions. Applying univariate ComBat independently to each metric fails to account for this shared structure, potentially leaving residual batch effects in cross-metric covariance.

To address this limitation, we extend the univariate ComBat (UV-ComBat) framework to a multivariate setting, which we refer to as the Multivariate ComBat (MV-ComBat) model. Building on the principles of existing ComBat methods, MV-ComBat assumes that, after removing fixed effects, the residuals follow a multivariate normal distribution, with independence across observations within each batch. This extension enables MV-ComBat to jointly model multiple correlated metrics rather than treating them separately, thereby allowing batch adjustment of both marginal distributions and the covariance structure across metrics.

#### 2.3.1 Multivariate Framework Setup

For simplicity, we introduce the multivariate ComBat framework under a linear model. For each batch i, feature v, and metric m, we assume

(1) $$Y_{ijvm}=\alpha_{vm}+X_{ijm}^{⊤}\beta_{vm}+Z_{ijvm},\;j=1,\ldots ,n_{i},$$

where $\alpha_{vm}$ is a metric-specific intercept, $X_{ijm}$ is the covariate vector, $\beta_{vm}$ are metric-specific fixed-effect coefficients, and $n_{i}$ is the number of subjects in batch i.

The residual term $Z_{ijvm}$ captures the batch-related variation. Stacking the residuals across metrics yields

$$Z_{ijv}={\left(Z_{ijv1},\ldots ,Z_{ijvM}\right)}^{⊤}\in R^{M},$$

which we assume follows a multivariate normal model,

$$Z_{ijv}\overset{\;\text{i.i.d.}\;}{∼}𝒩\left(\gamma_{iv},\Sigma_{iv}\right),$$

where $\gamma_{iv}\in R^{M}$ is the batch-specific location (mean) effect and $\Sigma_{iv}\in R^{M\times M}$ is a symmetric positive-definite covariance matrix capturing the batch-specific dispersion across the M metrics for feature v in batch i, and is shared across subjects j.

Under this framework, the essential difference from the univariate ComBat family is that residuals are modeled jointly across metrics. Specifically, for each feature v in batch i, residuals $Z_{ijv}$ follow a multivariate normal distribution with a batch-specific mean and covariance, enabling harmonization of cross-metric covariance.

#### 2.3.2 Estimating Batch Effect Parameters

To help with stable batch-effect estimation, we first use a regression-based approach to estimate and remove fixed effects. In the linear model, we compute the least-squares estimates ${\overset{ˆ}{\alpha }}_{vm}$ and ${\overset{ˆ}{\beta }}_{vm}$, and subtract the fitted values to obtain residuals $Z_{ijvm}$. Since the scale of metrics may differ across features and studies, these residuals are standardized to a common scale. To enable information sharing across features and metrics, batch effects are then estimated using two complementary Bayesian frameworks.

##### Empirical Bayes batch-effect estimation.

Similar to the univariate ComBat model, we adopt an empirical Bayes framework in which hyperparameters are estimated by MoM. To ensure conjugacy, we specify multivariate normal priors on the batch-specific means and inverse–Wishart (IW) priors on the batch-specific covariances:

$$\gamma_{iv}∼𝒩\left(\gamma_{i},T_{i}\right),\;\Sigma_{iv}∼IW\left(\Psi_{i},\nu_{i}\right).$$

For the multivariate normal likelihood, these priors yield closed-form conditional posteriors. Using MoM plug-in estimates $\left({\overset{ˆ}{\gamma }}_{i},{\overset{ˆ}{T}}_{i},{\overset{ˆ}{\Psi }}_{i},{\overset{ˆ}{\nu }}_{i}\right)$, the empirical-Bayes updates are:

(2) $$\gamma_{iv}^{*}=E\left[\gamma_{iv}|Z_{ijv},\Sigma_{iv}\right]={\left(n_{i}\Sigma_{iv}^{*-1}+{\overset{ˆ}{T}}_{i}^{-1}\right)}^{-1}\left(n_{i}\Sigma_{iv}^{*-1}{\bar{Z}}_{iv}+{\overset{ˆ}{T}}_{i}^{-1}{\overset{ˆ}{\gamma }}_{i}\right),\;{\bar{Z}}_{iv}=\frac{1}{n_{i}}\sum_{j=1}^{n_{i}}Z_{ijv},$$

and

(3) $$\Sigma_{iv}^{*}=E\left[\Sigma_{iv}|Z_{ijv},\gamma_{iv}\right]=\frac{\sum_{j=1}^{n_{i}}\;\left(Z_{ijv}\;-\;\gamma_{iv}^{*}\right){\left(Z_{ijv}\;-\;\gamma_{iv}^{*}\right)}^{⊤}\;+\;{\overset{ˆ}{\Psi }}_{i}}{n_{i}\;+\;{\overset{ˆ}{\nu }}_{i}\;-\;M\;-\;1}.$$

Detailed derivations are provided in Appendix A. We refer to this approach as MV-ComBat (EB). It provides a computationally efficient, closed-form solution that jointly shrinks both mean and covariance batch effects, borrowing strength across features and metrics while preserving fixed effects.

##### MCMC batch-effect estimation.

The empirical Bayes (MoM) approach is computationally efficient and performs well for point estimation in relatively low-dimensional settings, but it has several limitations: (i) it treats hyperparameters as fixed plug-in estimates and thus underestimates uncertainty; (ii) in high-dimensional settings, the IW prior can yield unstable or biased covariance estimates, as moment matching relies only on the diagonal second moments; and (iii) the IW prior links variances and correlations through a single degrees-of-freedom parameter, inducing undesirable dependence between marginal variances and correlations.

To address these limitations, we propose a fully Bayesian framework in which fixed effects are first removed by regression and batch effects are estimated via Hamiltonian Monte Carlo (HMC) with the No-U-Turn Sampler (NUTS), a *Markov Chain Monte Carlo (MCMC)* algorithm, implemented in Stan (Hoffman and Gelman 2014; Betancourt 2017; Stan Development Team 2024; Gabry et al. 2024) (Supplementary Figure S1). Since fully joint Bayesian estimation of regression and batch-effect parameters in high-dimensional multi-metric settings would substantially increase posterior dimensionality and computational cost, leading to poor MCMC mixing and limited scalability, we adopt this two-stage strategy to allow the Bayesian model to focus on batch-related mean and covariance estimation. To increase flexibility, we replace the IW prior with an LKJ prior (Lewandowski, Kurowicka, and Joe 2009) on the correlation matrix and independent half-*t* priors for the marginal standard deviations, thereby decoupling shrinkage of variances and correlations (Barnard, McCulloch, and Meng 2000). Posterior sampling proceeds by iteratively drawing from the joint posterior distribution of $\left(\gamma_{iv},\Sigma_{iv}\right)$. After convergence, posterior means are used as point estimates for $\gamma_{iv}^{*}$ and $\Sigma_{iv}^{*}$. We denote this approach as MV-ComBat (MCMC). Additional details of the multivariate Bayesian model are provided in Supplementary Section S1.

#### 2.3.3 Adjust the Data for Batch Effects

After estimating the batch-effect mean $\gamma_{iv}^{*}$ and covariance $\Sigma_{iv}^{*}$, MV-ComBat removes the batch-specific location and rescales the covariance, i.e.,

(4) $$Z_{ijv}^{*}=\Sigma_{iv}^{*-1/2}\left(Z_{ijv}-\gamma_{iv}^{*}\right),$$

where $Z_{ijv}^{*}={\left(Z_{ijv1}^{*},\ldots ,Z_{ijvM}^{*}\right)}^{⊤}\in R^{M}$, and $Z_{ijvm}^{*}$ denotes the adjusted, metric-specific component for subject j, feature v, and metric m. Intuitively, this transformation "whitens" the residual vector with respect to the estimated batch covariance, removing batch-induced cross-metric dependence so that residuals are on a common scale across batches.

Finally, the estimated fixed effects for each metric m are added back to obtain the harmonized observations:

(5) $$Y_{ijvm}^{\mathrm{ComBat}}={\overset{ˆ}{\alpha }}_{vm}+X_{ijm}^{⊤}{\overset{ˆ}{\beta }}_{vm}+Z_{ijvm}^{*}.$$

Beyond the linear-model setting, MV-ComBat extends naturally to generalized additive models (GAMs) and linear mixed models, providing multivariate extensions of ComBat-GAM and LongComBat.

### 2.4 Multivariate CovBat

As discussed above, MV-ComBat assumes independence of batch effects across features and may therefore struggle to correct strong batch effects in cross-feature covariance, particularly when these interact with underlying biological variation. To address this limitation, we extend the core concept of CovBat to a multivariate setting while preserving its two-stage harmonization framework. We refer to this approach as MV-CovBat.

Intuitively, whereas MV-ComBat adjusts batch effects within each feature’s cross-metric covariance, MV-CovBat additionally targets batch effects that persist in the cross-feature covariance structure by harmonizing a shared low-dimensional latent representation learned from all metrics.

#### Stage 1 (Standard MV-ComBat for feature-wise harmonization).

We first apply MV-ComBat to remove feature-wise batch effects in the mean and covariance across metrics. We then obtain the residuals for each metric, denoted as $R^{\left(m\right)}$, after removing fixed effects. Here $R^{\left(m\right)}\in R^{n\times p}$, where n is the number of subjects and p is the number of features (e.g., ROIs). To facilitate adjustment of batch effects in the covariance structure across features, we perform PCA on these residuals to transform them into orthogonal components, denoted as $F^{\left(m\right)}$:

$$R^{\left(m\right)}=F^{\left(m\right)}L^{(m)⊤},\;F^{\left(m\right)}\in R^{n\times r_{m}},L^{\left(m\right)}\in R^{p\times r_{m}},$$

where $r_{m}$ is the number of principal components (PCs) retained to explain most of the variation (with a default of 95%), n is the number of observations per metric, and p is the number of features within each metric. Here $F^{\left(m\right)}$ contains subject (score) vectors and $L^{\left(m\right)}$ contains the corresponding feature loading vectors.

We assume that there exists a common low-dimensional latent space G that explains variation shared across all metric-specific PCA spaces:

$$F^{\left(m\right)}=GA^{\left(m\right)}+H^{\left(m\right)},$$

where $G\in R^{n\times r_{s}}$ represents the shared subject scores across all metrics, $r_{s}$ denotes the dimensionality of the shared latent subspace capturing common variation, $A^{\left(m\right)}\in R^{r_{s}\times r_{m}}$ is the projection from the shared to the metric-specific subspace, and $H^{\left(m\right)}$ is an idiosyncratic component unique to metric m.

#### Stage 2 (Latent space harmonization).

The main goal of the second stage is to estimate the shared latent space G and harmonize it to remove potential batch effects in covariance across features and, indirectly through sharing, across metrics. Without loss of generality, we impose an orthonormality constraint on G.

We estimate G and the $A^{\left(m\right)}$ by solving

(6) $$\underset{G,\{A^{\left(m\right)}\}}{min}\;\sum_{m=1}^{M}{\left\|F^{\left(m\right)}-GA^{\left(m\right)}\right\|}_{F}^{2}\;\;\text{s.t.}\;G^{⊤}G=I_{r_{s}}.$$

Here M denotes the total number of metrics, $F^{\left(m\right)}\in R^{n\times r_{m}}$ contains metric-specific PCA scores, $G\in R^{n\times r_{s}}$ contains shared subject scores, and $A^{\left(m\right)}\in R^{r_{s}\times r_{m}}$ maps the shared space to the metric-specific PCA score.

Fixing G, the least-squares solution is $A^{\left(m\right)}=G^{⊤}F^{\left(m\right)}$. Let $P=GG^{⊤}$ denote the orthogonal projection matrix onto the column space of G. Substituting $A^{\left(m\right)}$ back yields:

$$F^{\left(m\right)}-GA^{\left(m\right)}=\left(I-P\right)F^{\left(m\right)}.$$

By the Pythagorean theorem, the objective function becomes:

(7) $$\sum_{m=1}^{M}{\left\|(I-P)F^{\left(m\right)}\right\|}_{F}^{2}=\sum_{m=1}^{M}{\left\|F^{\left(m\right)}\right\|}_{F}^{2}-\sum_{m=1}^{M}{\left\|G^{⊤}F^{\left(m\right)}\right\|}_{F}^{2}=const-Tr\left(G^{⊤}\left[\sum_{m=1}^{M}\;F^{\left(m\right)}F^{\left(m\right)⊤}\right]G\right).$$

Thus, minimizing the objective is equivalent to maximizing $Tr\left(G^{⊤}SG\right)$, where $S=\sum_{m=1}^{M}F^{\left(m\right)}F^{(m)⊤}$. By the Rayleigh–Ritz theorem, the maximizer G consists of the top $r_{s}$ eigenvectors of S.

We then apply standard univariate ComBat using a linear model to the columns of G, treating subjects as observations and latent dimensions as features, to remove batch effects in the mean and scale of the shared latent scores. The harmonized latent scores are subsequently propagated back to the metric-specific spaces via the reconstruction described above, thereby correcting covariance-related batch effects across features and metrics.

### 2.5 Simulation design

We first evaluate MV-ComBat against UV-ComBat under two simulation scenarios. The first, denoted *model-concordant*, generates batch effects according to the EB assumptions of MV-ComBat, producing an unstructured covariance distribution that aligns with the proposed model. The second, denoted *model-misspecified*, draws batch-specific covariances from a mixture of distributions, including strongly structured covariance patterns such as autocorrelation (AR), compound symmetry (CS), and factor-analytic (FA) structures, thereby inducing structural mismatch with the exchangeable covariance prior assumed in MV-ComBat. These two scenarios are designed to assess the robustness of the MV-ComBat framework under both correctly specified and misspecified covariance priors.

Within each scenario, we examine three experimental conditions: two regular conditions with mild batch effects and different sample sizes, and one *stress-test* condition with severe batch effects. The regular conditions represent realistic data settings, whereas the stress test evaluates performance under extreme conditions, thereby assessing the overall robustness of MV-ComBat.

#### Data-generating scheme.

We first generate baseline values that contain only biologically meaningful variation from prespecified covariates. We consider I=3 batches, p=70 features, M=6 metrics per feature, and three sample sizes (n=100,150,500). Biological variability is introduced through three covariates: age, sex, and diagnosis.

For each subject j, we simulate ${Age}_{j}∼𝒩\left(50,10^{2}\right)$ and ${Sex}_{j}∼Bernoulli(0.5)$. For diagnosis, we draw a binary indicator $D_{j}∼Bernoulli(0.3)$ and select a biomarker subset ℬ⊂{1,…,p} of size 0.6p (rounded to the nearest integer). Diagnosis effects are applied only to features in ℬ, with weaker effects introduced in the first two metrics and stronger effects in the remaining four. We then add linear covariate effects to each feature v=1,…,p and metric m=1,…,M:

$$Y_{jvm}^{\text{base}\;}=\epsilon_{jvm}+\beta_{vm}^{\text{Age}\;}{Age}_{j}+\beta_{vm}^{\text{Sex}\;}{Sex}_{j}+\beta_{vm}^{\text{Diag}\;}D_{j}I\{v\in ℬ\},$$

where the baseline values $Y_{jvm}^{\text{base}}$ serve as the gold standard against which we evaluate harmonization performance, particularly with respect to the recovered correlation structure.

#### Feature-wise batch effects.

For each batch i and feature v, we first draw a location shift $\gamma_{iv}\in R^{M}$ as $\gamma_{iv}∼𝒩\left(\gamma_{i},T_{i}\right)$. We then generate the batch–feature covariance $\Sigma_{iv}$ using one of two schemes: (1) from an IW distribution for the model-concordant scenario, and (2) from a mixture of covariance families (IW, LKJ, FA, AR, CS) with weights w=(0.20,0.30,0.20,0.20,0.10) for the model-misspecified scenario.

Finally, for each subject j in batch i, we generate unharmonized data as

$$Y_{ijv}^{\text{unharm}}=Y_{ijv}^{\text{base}}+\gamma_{iv}+\varepsilon_{ijv},\;\varepsilon_{ijv}\overset{\;\text{i.i.d.}\;}{∼}𝒩\left(0,\Sigma_{iv}\right).$$

This process yields unharmonized data containing batch effects. The magnitudes of covariate and batch effects vary across the three experimental conditions. Detailed parameter settings are provided in Supplementary Tables S1 and S2. We perform R=100 independent replicates per scenario to evaluate performance.

#### Latent-space batch effects.

To further evaluate the performance of MV-CovBat and assess the robustness of MV-ComBat under structured, low-rank batch variation, we introduce batch effects through perturbations in a shared latent space:

$$G_{i}^{\left(m\right)}=G_{i}R_{m}+\mu_{m}+E_{im},\;E_{im}∼𝒩\left(0,\Sigma_{G,m}\right),$$

where $R_{m}\in R^{r\times r}$ is a batch-specific affine rotation that induces correlated distortions across metrics, $\mu_{m}\in R^{1\times r}$ is a mean-shift vector introducing low-rank batch mean differences, and $\Sigma_{G,m}$ is a batch-specific latent covariance controlling residual variability. The resulting $G_{i}^{\left(m\right)}$ generates batch effects across both features and metrics through the feature loading matrices $A^{\left(m\right)}$ (as described in the Multivariate CovBat section), thereby inducing multivariate batch distortions that challenge MV-ComBaťs ability to recover the underlying covariance structure.

We apply this latent batch-effect mechanism in two settings: (1) to simulated data following the same data-generating scheme as above, but with varying sample sizes, feature numbers, and diagnosis effect magnitudes, including additional AR(1)-correlated diagnosis effects (Controlled Simulation); and (2) to the A2CPS dataset, restricting to a single site (UI–GE) to introduce controlled batch perturbations (Empirical Simulation).

These experiments allow us to assess how different harmonization methods perform in removing covariance-level batch effects across features while preserving genuine biological variation, particularly when common assumptions about batch distributions are violated.

## 3 Harmonization Evaluation Methods

We evaluated harmonization performance across three complementary aspects: (1) batch removal, (2) biological signal preservation, and (3) feature-wise correlation recovery.

Feature-wise batch removal was assessed using univariate tests for mean differences, including ANOVA and the Kruskal–Wallis test (Kruskal and Wallis 1952), and tests for variance and scale differences, including Levene’s test (Levene et al. 1960), Bartlett’s test (Bartlett 1937), and the Fligner–Killeen test (Fligner and Killeen 1976). We additionally performed multivariate assessments using MANOVA to evaluate differences in multivariate location and Box’s M test (BOX 1953) to evaluate differences in covariance across batches and metrics. All p-values were Bonferroni-corrected. Residual global batch effects were further evaluated using stratified 10-fold cross-validated random forest classifiers to predict site/scanner (R packages caret and randomForest; ntree=100, other hyperparameters at defaults), with macro-averaged area under the ROC curve (AUC) quantifying residual batch-related variation.

To evaluate preservation of biologically meaningful signal, we fit feature-wise regression models using prespecified covariates and counted the number of significant features. For the A2CPS dataset, where the true biological effects are unknown, we controlled the false discovery rate (FDR) using the Benjamini–Hochberg (BH) procedure. In simulation studies, where the truth was known, we reported the number of true-positive discoveries and the empirical FDR to assess accuracy in recovering true associations.

We evaluated correlation recovery by comparing within-metric and cross-metric (pooled feature) correlations to the ground truth in simulations. Similarity between estimated and true correlation structures was quantified using four matrix-distance metrics: the Frobenius norm and element-wise MSE for overall recovery, and eigenvalue error and spectral norm for detecting structural distortions. Lower values indicate better recovery. These metrics were also used to assess batch-related differences in correlation matrices across batch levels, with smaller values reflecting weaker batch effects.

Finally, for the EB approach, we evaluated model assumptions by comparing empirical distributions of estimated additive batch effects with their prior distributions. For multiplicative effects, we performed prior predictive checks by comparing empirical covariance matrices with draws simulated from the IW prior. Matrix-distance metrics quantified deviations from the prior mean, and histograms of these statistics visualized empirical–prior agreement. Substantial overlap indicated that the EB assumptions were reasonably satisfied. For the fully Bayesian approach, convergence of the Markov chains was assessed using standard diagnostics, including the potential scale reduction factor ($\overset{ˆ}{R}$), effective sample size, and visual inspection of trace plots, with $\overset{ˆ}{R}<1.01$ used as the convergence criterion.

## 4 Results

### 4.1 A2CPS Study

In the A2CPS study, we considered age, sex, and pre-surgical pain duration as biologically meaningful sources of variation that should be preserved after harmonization. The sample demographics were well balanced across sites, which minimized confounding between these covariates and batch effects and supported their preservation in the harmonization process.

#### Regression Model Specification.

**Figure 1:**
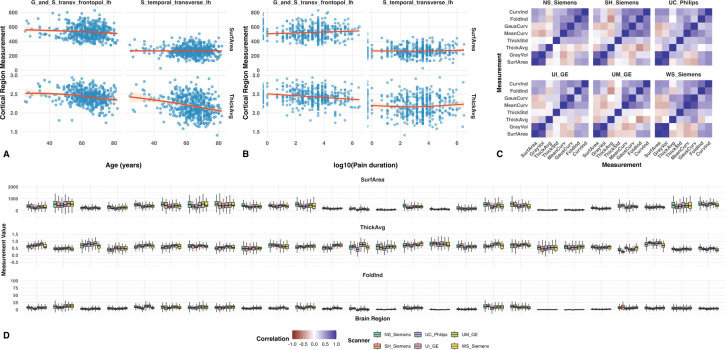
Exploratory Analysis. (A–B) Scatterplots of cortical-region metrics against age and pre-surgical pain duration show nonlinear associations, motivating the use of generalized additive models (GAMs) for covariate adjustment. (C) Residual cross-metric correlation matrices, stratified by batch, show strong correlations among some metrics and distinct correlation patterns across sites, consistent with scanner-related effects not captured by fixed effects. (D) Residual distributions by site, highlighting additive and multiplicative batch effects across regions and metrics. Some metrics (e.g., FoldInd) exhibit influential outliers in the underlying data, which affect the scale.

Exploratory plots of cortical-region metrics versus age and pain duration suggested nonlinear associations (Figure 1A,B). We therefore fit generalized additive models (GAMs) to estimate fixed effects to be preserved during harmonization. Sex was included as a parametric term, while age and pre-surgical pain duration were modeled as smooth functions. As shown in Figure 1C, strong residual cross-metric correlations persisted among some metrics, and the correlation patterns differed across batch levels, suggesting scanner-related effects not captured by the initial model and retained in the cross-metric covariance. Residual distributions, stratified by site, indicated both additive (mean-shift) and multiplicative (scale) batch effects across all cortical metrics, with magnitudes varying by ROI and metric. We also observed influential outliers in some metrics, such as FoldInd, which may pose challenges for ComBat-type methods (Figure 1D).

#### Feature-wise Batch Detection.

We compared five harmonization methods: (1) MV-ComBat (EB), (2) MV-ComBat (MCMC), (3) UV-ComBat, (4) MV-CovBat, and (5) UV-CovBat, using GAMs to estimate fixed effects. For simplicity, only the EB variant was applied to both CovBat methods. Overall, the EB assumptions for MV-ComBat (EB) were well met for this dataset (Supplementary Figure S2). Additionally, for the MCMC model, all parameters exhibited $\overset{ˆ}{R}<1.01$ and effective sample sizes exceeding 400, indicating satisfactory convergence to the target posterior distribution. Feature-wise batch-effect diagnostic results were then compared across the five harmonization methods as well as the unharmonized data.

**Figure 2:**
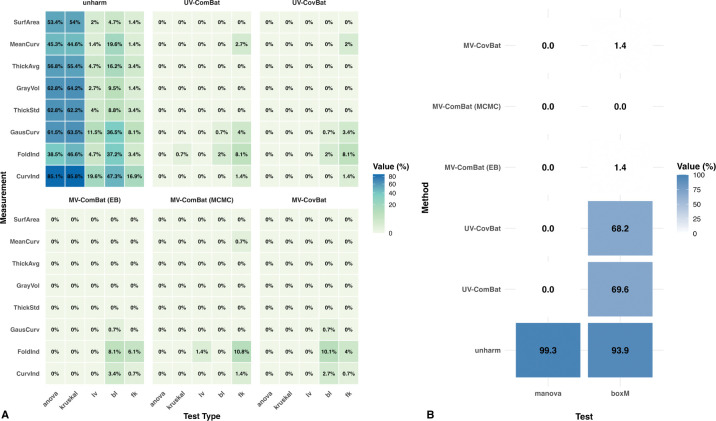
Statistical Tests. (A) Univariate statistical tests within each metric comparing (1) unharmonized data, (2) UV-ComBat, (3) UV-CovBat, (4) MV-ComBat (EB), (5) MV-ComBat (MCMC) and (6) MV-CovBat with GAM-based fixed effects preserved. Additive effects are tested using ANOVA and Kruskal–Wallis; multiplicative effects using Levene’s, Bartlett’s, and Fligner–Killeen. Both MV-ComBat variants outperform UV-ComBat for MeanCurv and GausCurv, but not for CurvInd and FoldInd where influential outliers limit gains. Within MV-ComBat, EB slightly exceeds MCMC for MeanCurv, while MCMC is better for GausCurv and CurvInd. (B) Multivariate statistical tests of location and covariance across metrics (MANOVA and Box’s M). UV-ComBat leaves substantial covariance differences, whereas MV-ComBat markedly reduces them. In particular, MV-ComBat (MCMC) effectively removes covariance batch effects. UV-CovBat slightly outperformed UV-ComBat.

Univariate test results (Figure 2A) showed that both MV-ComBat methods outperformed UV-ComBat for MeanCurv and GausCurv, but not for CurvInd and FoldInd, likely reflecting the influence of outliers. The EB variant performed better for MeanCurv, while MCMC performed better for GausCurv and CurvInd. Multivariate tests (Figure 2B) revealed that UV-ComBat failed to remove batch effects in covariance across metrics, with 70% of ROIs showing significant differences, whereas both MV-ComBat methods substantially reduced these effects, leaving no ROIs with significant covariance differences for the MCMC variant after correction. Comparing the ComBat and CovBat frameworks (in both MV and UV settings), we found that they performed comparably in both univariate and multivariate analyses. Together, these results indicate that joint modeling substantially improves feature-wise cross-metric covariance harmonization.

#### Fixed-effects Preservation and Global Batch Detection.

**Figure 3:**
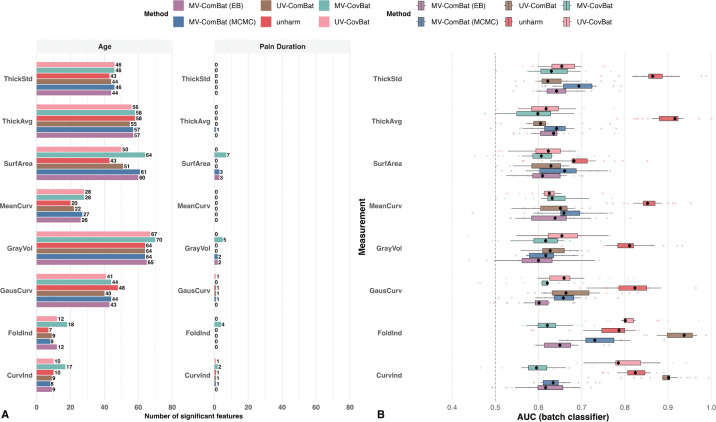
Detection of Batch Effects and Biological Variation. (A) Number of ROIs with significant nonlinear fixed effects from GAMs fit to unharmonized data and to data harmonized by MV-ComBat (EB), MV-ComBat (MCMC), UV-ComBat, MV-CovBat, and UV-CovBat. Across most metrics, MV-ComBat preserves at least as many signals as UV-ComBat, with substantial gains for SurfArea. Pain duration shows weaker effects overall. Both UV-CovBat and MV-CovBat identified more significant signals than their ComBat counterparts, with MV-CovBat detecting substantially more. (B) Residual batch signal measured by 10-fold CV random-forest AUC for predicting site/scanner from features. Lower AUC indicates better batch removal. MV-CovBat yields the lowest AUCs for most metrics. UV-CovBat outperforms UV-ComBat but still shows high AUCs for CurvInd and FoldInd, indicating poor batch removal likely driven by severe outliers. MV-ComBat (MCMC) is slightly less effective than MV-ComBat (EB) in most metrics, but generally outperforms UV-ComBat and is more robust when outliers are present.

We next assessed whether harmonization preserved nonlinear covariate effects using GAMs and whether residual global batch effects remained within each metric. Age effects were consistently strong, while pain duration effects were considerably weaker. Notably, in GausCurv, the unharmonized dataset yielded more significant findings, some of which may reflect residual batch effects. That is, a decrease in the number of significant findings does not necessarily imply a loss of true signal. After controlling FDR, MV-ComBat generally retained as many or more signals than UV-ComBat, and substantially more signals for SurfArea (Figure 3A). We therefore conclude that MV-ComBat preserved more putative biological signals overall.

Evaluating global batch signals within each metric, the random-forest classifier showed that UV-ComBat yielded slightly lower AUCs for ThickStd and ThickAvg, but most values were below 0.7 for all methods, indicating weak residual batch signals. For other metrics, MV-ComBat (EB) performed best among the ComBat methods, whereas UV-ComBat exceeded 0.9 for CurvInd and FoldInd, reflecting its failure to remove batch effects in the presence of severe outliers (Figure 3B). MV-ComBat (MCMC) was slightly less effective than the EB variant in most metrics, likely due to prior misspecification and cross-metric borrowing, but still outperformed UV-ComBat, particularly in outlier-prone metrics.

Extending to covariance harmonization, both UV-CovBat and MV-CovBat detected more significant signals than their ComBat counterparts, with MV-CovBat showing the greatest gains. Although UV-CovBat outperformed UV-ComBat in outlier-prone metrics, the AUCs for UV-CovBat remained high (around 0.8), indicating that considerable batch effects persisted. Compared with MV-ComBat (EB), MV-CovBat further improved correction in FoldInd, CurvInd, ThickAvg, and ThickStd, achieving the best performance in global batch removal. Taken together, these results indicate that the CovBat frameworks outperformed the ComBat frameworks in removing residual global batch signals present in feature values and their correlations at the metric level, thereby improving the detection of biologically meaningful signals, with MV-CovBat achieving the strongest overall performance.

#### Batch Detection in Correlation.

**Figure 4:**
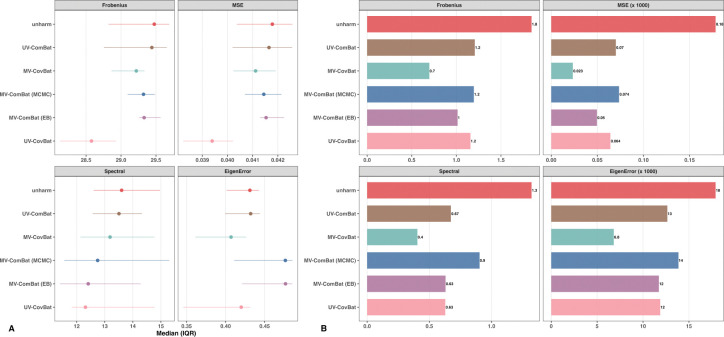
Batch Detection in Correlation. (A) Average within-metric correlation matrix distance across batch levels. Points indicate medians across ROIs, and horizontal error bars denote the interquartile range (IQR; 25th–75th percentiles). UV-CovBat performs best overall, while MV-CovBat outperforms both MV-ComBat variants. (B) Average cross-metric correlation matrix distance across batch levels. MV-CovBat performs best overall. For visualization, MSE (×1000) and EigenError (×1000) indicate that values were multiplied by 1000 to improve scale comparability.

Finally, we evaluated batch removal in correlations by comparing the average distance between pairwise correlation matrices across batches using the four previously described distance metrics. UV-CovBat performed best for within-metric correlations, which is consistent with its single-metric harmonization design. MV-CovBat outperformed MV-ComBat in all metrics except the spectral norm (Figure 4A), indicating strong correction with minimal structural distortion. For cross-metric correlations, MV-CovBat achieved the most effective batch removal (Figure 4B), highlighting its advantage in jointly modeling batch effects across metrics and performing latent space harmonization.

### 4.2 Simulation

#### Feature-wise Batch-effect Simulation.

To better understand the performance of MV-ComBat in removing feature-wise batch effects across metrics, we applied (i) MV-ComBat (EB), (ii) MV-ComBat (MCMC), and (iii) UV-ComBat to simulated data, preserving age, sex, and diagnosis effects using linear models. Harmonization performance was evaluated under three experimental conditions within the model-concordant scenario, with additional results under model misspecification reported to assess the robustness of the proposed methods.

#### Batch removal.

Following the A2CPS study, we assessed within-metric global batch effects using 10-fold cross-validated random forest AUC. All three methods substantially reduced batch effects across the three experimental conditions in both scenarios, with mean AUCs for batch detection remaining around 0.6 for smaller sample sizes and further decreasing to 0.56 for the largest sample size (n = 500) (Table 1; Supplementary Table S3). Accordingly, within-metric AUCs were similar across methods. This is expected because the evaluation focuses on within-metric marginal batch effects, a setting in which UV-ComBat is specifically designed to perform well and therefore performs comparably to MV-ComBat in this global batch-effect setting.

In Box’s M test, under the model-concordant scenario, about 25% and 60% of features in the unharmonized dataset showed covariance-related batch effects in the regular conditions with sample sizes of 150 and 500, respectively, and roughly 30% in the stress test. After UV-ComBat, these proportions decreased to approximately 12% and 37% in the regular conditions but increased to 50% in the stress test.

**Figure 5:**
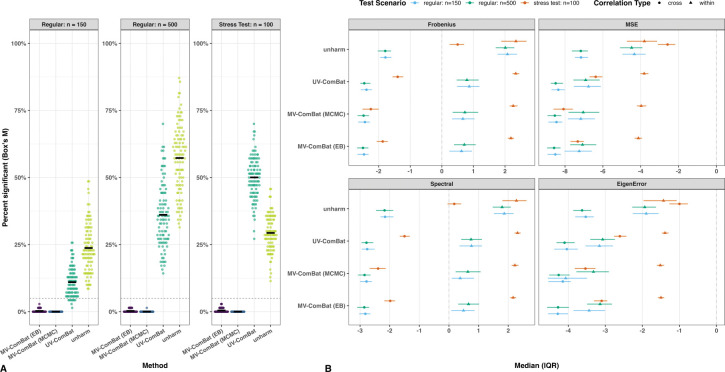
Batch Detection in Covariance and Correlation Recovery. (A) Box’s M test across experimental conditions. Both MV-ComBat variants outperformed UV-ComBat, with the MCMC variant showing greater gains in smaller samples. (B) Correlation recovery. The MCMC approach achieved lower eigenvalue error for within-metric correlations under regular conditions with small samples and better cross-metric recovery under stress when priors were correctly specified (for misspecification results, seep Supplementary Figure S3).

In contrast, both MV-ComBat variants produced datasets largely free of covariance-related batch effects, with the MCMC variant performing particularly well (Figure 5A). Under the model-misspecified scenario, results were similar, except that MCMC remained robust under mild batch effects but was occasionally less stable in the stress test due to prior mismatch (Figure S3A).

Overall, these findings highlight a key strength of MV-ComBat: its ability to mitigate batch effects in cross-metric covariance. By comparison, UV-ComBat effectively removed within-metric batch effects but struggled to address covariance-related effects across metrics.

##### Correlation Recovery.

Under the model-concordant scenario, cross-metric correlations generally showed smaller deviations from the gold standard than within-metric correlations, reflecting the clean, homoscedastic structure of the simulated data (Figure 5B). For within-metric recovery, both MV-ComBat variants outperformed UV-ComBat across all experimental conditions. The MCMC variant showed substantially better eigenvalue error performance at a sample size of 150, although this advantage diminished with larger samples. In the stress test, all methods performed similarly, with the EB variant showing slightly better performance, potentially indicating greater robustness. For cross-metric correlations, both MV-ComBat variants markedly outperformed UV-ComBat in the stress test, with the MCMC variant performing best.

Under model misspecification (Supplementary Figure S3B), results were largely consistent with the model-concordant scenario. However, the advantage of the MCMC variant over the EB variant for cross-metric recovery in the stress test disappeared, likely due to prior misspecification. Overall, MV-ComBat (MCMC) exhibited stronger performance in preventing within-metric correlation distortion (especially in small samples) and in recovering cross-metric correlations when batch effects were extremely strong and priors were correctly specified. The MCMC approach was robust to prior misspecification under mild batch effects, whereas the EB variant was more robust to model misspecification.

##### Signal Preservation.

**Figure 6:**
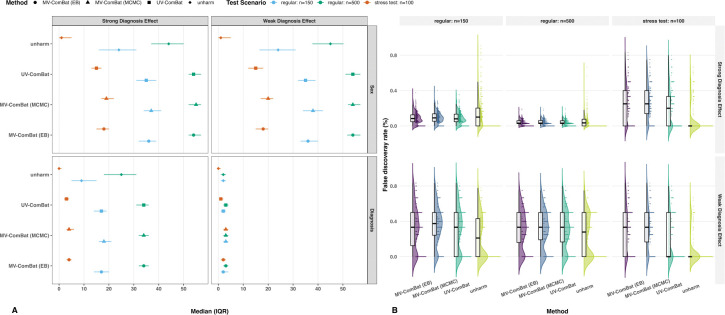
Fixed-effects Detection and Corresponding FDRs. (A) Preservation of biological signals. Counts of features with significant sex and diagnosis effects across experimental conditions. MV-ComBat (MCMC) generally preserved the most fixed effects, with greater advantages in smaller samples, under stress, and for moderate-to-strong signals. (B) False discovery rate (FDR) based on known biomarkers. Both MV-ComBat variants performed comparably to UV-ComBat, although UV-ComBat showed lower mean FDRs in the stress test due to missed detections rather than improved specificity (for misspecification results, see Supplementary Figure S4).

We fitted linear models to assess age, sex, and diagnosis effects. As designed, all ROIs showed strong age effects and moderate-to-strong sex effects, while 60% served as biomarkers with weak diagnosis effects in the first two metrics and moderate-to-strong effects in the remaining ones. We focused on weak and moderate-to-strong signals, as these are more likely to be removed along with batch effects under confounding. MV-ComBat (MCMC) generally preserved the most fixed effects while maintaining FDRs comparable to UV-ComBat. Its advantages were most pronounced in smaller samples, under the stress test, and for moderate-to-strong signals (Figure 6A). FDR decreased with increasing sample size and stronger signals (Figure 6B). In the stress test, UV-ComBat and unharmonized data exhibited lower mean FDRs due to missed detections that artificially reduced averages. Thus, the slightly higher FDRs observed with MV-ComBat should not raise concern, as they likely reflect increased sensitivity rather than inflated false positives. Under model misspecification, results were similar, though the MCMC advantage over EB diminished due to prior misspecification (Supplementary Figure S4). Overall, MV-ComBat achieved superior preservation of moderate-to-strong biological signals.

#### Latent-space Batch-effect Simulation.

As noted earlier, MV-ComBat assumes independent batch effects across features and may therefore struggle when strong batch effects remain in feature correlations. To evaluate whether MV-CovBat addresses this limitation, we applied: (1) MV-ComBat, (2) MV-CovBat, (3) UV-ComBat, and (4) UV-CovBat (all using the EB approach) to the Controlled Simulation (linear model preserving age, sex, and diagnosis) and to the Empirical Simulation (GAM preserving age, sex, and pain duration). In this section, we focus on two key aspects: (1) removal of batch effects in feature correlations and (2) preservation of fixed effects measured by FDR, as these represent the main advantages of the CovBat framework.

##### Controlled Simulation.

**Figure 7:**
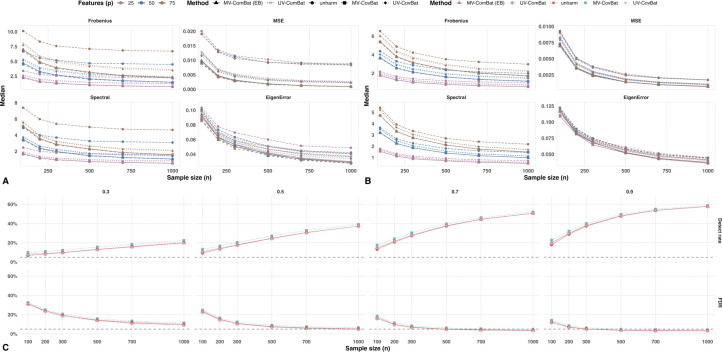
Batch Detection in Correlation and Diagnosis Effect Preservation. (A) Average within-metric correlation matrix distance across batch levels. Both MV-CovBat and MV-ComBat outperform UV-CovBat and UV-ComBat, with MV-CovBat showing greater improvement, particularly in small sample sizes and large feature sets. (B) Average cross-metric correlation matrix distance across batch levels. MV-CovBat continues to perform best, with minimal differences from MV-ComBat. (C) Detection of diagnosis effects and corresponding FDRs. MV-CovBat consistently identifies more true signals, with slightly higher FDRs. When the diagnosis effect size is 0.9 and the sample size is 500, the FDR for MV-CovBat drops below 0.05.

We calculated the average pairwise distances between correlation matrices across batch levels for both within- and cross-metric correlations using the four matrix-distance criteria. For within-metric correlations, MV-CovBat consistently achieved the smallest distances across all criteria, particularly in smaller samples with more features (Figure 7A). As sample size increased, the gap between MV-CovBat and MV-ComBat narrowed. Both multivariate methods (MV-ComBat and MV-CovBat) outperformed their univariate counterparts across all sample sizes and feature counts.

For cross-metric correlations, we observed a similar pattern, though the advantage of MV-CovBat over MV-ComBat was smaller (Figure 7B), consistent with the simulation design in which cross-metric batch effects were relatively weak and homogeneous across batches. In terms of fixed-effect preservation, MV-CovBat consistently detected more true signals, accompanied by slightly higher FDRs (Figure 7C), reflecting the common power–FDR trade-off. Differences in FDR across methods were minimal, and as sample size and effect size increased, all methods detected more diagnosis effects while maintaining lower FDRs. With moderate-to-strong diagnosis effects ($\beta_{d}=0.9$) and a median sample size (n=500), the FDR for MV-CovBat remained below 0.05. These results highlight MV-CovBat’s strength in preserving genuine biological signals, likely facilitated by improved correction of correlation-related batch effects.

##### Empirical Simulation.

**Figure 8:**
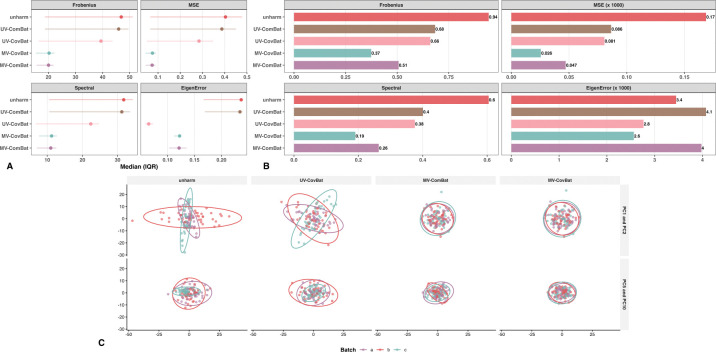
Batch Detection in Correlation. (A) Average within-metric correlation matrix distance across batch levels. MV-CovBat performs comparably to MV-ComBat and substantially better than both univariate methods. (B) Average cross-metric correlation matrix distance across batch levels. MV-CovBat shows the best overall performance. (C) PCA of the *G* space. Residuals after UV-CovBat display pronounced directional variation, indicating confounding between batch levels and shared latent scores. In contrast, residuals after MV-ComBat and MV-CovBat form more homogeneous clusters in the first two components, with MV-CovBat performing better in higher-order components.

We randomly assigned three batch levels to the single-site subset of the A2CPS data (130 observations) and introduced latent batch effects following the same procedure as in the controlled simulations. MV-ComBat performed comparably to or slightly better than MV-CovBat in removing within-metric batch effects, likely due to its greater robustness to outliers (Figure 8A). Both methods, however, substantially outperformed UV-CovBat and UV-ComBat. The PCA plot of the *G* space (Figure 8C) revealed potential confounding between batch levels and shared latent scores after UV-CovBat, as indicated by pronounced directional variation within clusters. Both MV-ComBat and MV-CovBat greatly reduced these directional differences in the first two principal components, with MV-CovBat performing better in the higher-order components. Moreover, MV-CovBat achieved superior performance in pooled correlations across metrics for all four criteria (Figure 8B), highlighting its strength in correcting cross-feature and cross-metric covariance distortions that arise under strong multivariate batch effects.

## 5 Discussion

Our findings highlight the importance of robust harmonization for multi-site and multimodal neuroimaging data. While univariate methods can effectively mitigate site effects within individual metrics, they do not address covariance across metrics, an aspect crucial for multimodal analyses in which biological processes are inherently correlated. In addition, univariate methods can underperform in settings where data are noisy or contain severe outliers. To overcome these limitations, we developed two complementary multivariate frameworks: MV-ComBat, which jointly harmonizes correlated metrics to adjust for batch effects in means, variances, and covariances, and MV-CovBat, which extends this framework through latent-space harmonization to remove residual covariance-related batch effects. Together, these methods enhance the preservation of biologically meaningful variation and the recovery of cross-metric relationships.

Several cortical metrics considered in the A2CPS study are mathematically or biologically related (e.g., mean curvature and folding index), resulting in a high-dimensional setting with strong inter-feature correlations. Such structure poses nontrivial challenges for harmonization methods, particularly those that do not explicitly account for covariance across features. In practice, large-scale neuroimaging studies often analyze collections of derived measures that differ in scale, variability, and interpretability, and whose relationships may not be fully characterized *a priori*. Accordingly, the A2CPS dataset represents a realistic and challenging application setting for evaluating harmonization methods under conditions commonly encountered in contemporary multi-site neuroimaging consortia.

In the A2CPS data, we showed that substantial batch effects exist not only in mean and variance within each cortical region metric, but also in the covariance across ROIs and metrics. We also identified influential outliers in some metrics, likely due to measurement errors, which can challenge the performance of the standard ComBat framework (Figure 1). These observations are consistent with prior studies highlighting the limitations of univariate harmonization for correlated neuroimaging features, including substantial discrepancies in covariance matrices even after univariate site correction and particularly poor reliability for cortical thickness due to high measurement error (Carmon et al. 2020). Together, these findings underscore the need for harmonization methods that explicitly model cross-metric dependence.

Using the A2CPS data, we further demonstrated that MV-ComBat outperforms UV-ComBat in removing batch signals within each metric that remain detectable by machine learning–based methods, particularly in the presence of significant measurement error, by borrowing information across metrics (Figure 3B). In addition, both multivariate and univariate CovBat frameworks outperformed their ComBat counterparts, with MV-CovBat achieving the best overall performance, highlighting its strength in removing residual cross-feature batch signals. Between the two batch estimation methods, the EB variant was more robust to measurement error in this dataset. As a direct benefit of batch effect removal, both MV-ComBat and MV-CovBat preserved more biological signals than the univariate framework, particularly MV-CovBat, while controlling the false discovery rate (FDR) using the Benjamini–Hochberg (BH) procedure (Figure 3A).

In our simulations with feature-wise batch effects, both variants of MV-ComBat effectively reduced batch effects in the covariance across features and metrics and preserved more biological variation across a range of effect sizes, though at the cost of slightly higher FDR when signals were weak (Figure 6). The MV-ComBat framework also demonstrated strong performance in recovering correlation structure across both features and metrics. It achieved the greatest improvements in recovering cross-metric correlations, particularly when batch effects were strong (Figure 5B). Importantly, although MV-ComBat assumes that batch effects are independent across features, we still observed improved recovery of cross-feature correlations. This improvement likely arises as an indirect benefit of borrowing information across metrics, which stabilizes feature-wise batch-effect estimates and mitigates spurious correlations induced by site and scanner effects.

When comparing the two estimation variants, the MCMC approach outperformed the EB variant in preventing within-metric correlation distortion under mild batch effects with small sample sizes. It also performed better in recovering cross-metric correlations under strong batch effects when priors were correctly specified. Notably, the MCMC variant retained strong performance even with misspecified priors under mild batch effects, likely because the likelihood from relatively clean data dominated and the Bayesian framework more effectively captured posterior uncertainty. However, under more severe batch effects, the EB variant was more robust, whereas the MCMC approach became less stable and occasionally failed due to prior misspecification (Supplementary Figure S3). These failures were largely attributable to using weakly informative priors to model strongly structured covariance structures or mixtures of heterogeneous covariance regimes across features.

In the MCMC model, we used a separation strategy with half-*t* priors on marginal scales and an LKJ prior on the correlation matrix, yielding an unstructured yet stable prior for the covariance matrix. When batch covariances were generated from an IW distribution, which is known to produce unstructured covariance, our chosen priors aligned well with the true structure, substantially improving covariance recovery through multivariate pooling. In contrast, when covariances arose from structured families such as autoregressive (AR) or compound symmetry (CS), the exchangeable LKJ prior misrepresented the underlying patterns and over-shrank correlations toward independence (Supplementary Figure S6). Additionally, when covariances were drawn from a mixture of heterogeneous covariance structures across features, the LKJ prior further imposed an implicit exchangeability assumption, encouraging overly similar correlation patterns across features and attenuating feature-specific structure. Under such prior–likelihood mismatches, particularly in settings with limited sample size or strong batch effects, posterior inference became strongly regularized by the prior, leading to under-recovery of covariance structure or inflated Type I error rates. To address this limitation and enhance flexibility across diverse covariance structures, future extensions of MV-ComBat should incorporate more flexible covariance priors capable of adapting to both unstructured and structured dependence patterns, as well as mixtures of heterogeneous covariance structures across features.

In simulations with latent-space batch effects, MV-CovBat showed superior performance in removing batch effects in feature correlations, both within and across metrics, thereby improving separation of biologically meaningful signals (Figure 8A). Although MV-ComBat performed slightly worse than MV-CovBat in addressing cross-metric correlations, it remained robust in mitigating batch effects in feature correlations, even when the assumed batch-effect distribution was violated. This robustness stems from its multivariate modeling, which borrows information across metrics and facilitates cross-feature correlation recovery, even when batch effects strongly interact with biological signals. In contrast, UV-CovBat performed poorly under strong confounding between batch and covariate effects, as treating each metric independently limited its ability to disentangle the signals (Figure 8B). These results highlight another key advantage of the multivariate framework.

Despite these advantages, several limitations remain. Like the original ComBat framework, our method requires pre-specifying covariates to preserve biologically meaningful signals. Prior work has noted that ComBat-based harmonization cannot preserve the effects of covariates that are not explicitly included in the model, particularly when those covariates are confounded with site (Bayer et al. 2022 ). Consequently, omitting a relevant covariate may result in its associated variation being inadvertently removed along with batch effects. We compared harmonization performance in preserving an omitted covariate across univariate and multivariate frameworks using both the A2CPS dataset, which provides a realistic scenario with substantial confounding, and simulations in which the omitted covariate was designed to have nonconfounding effects relative to batch. We found that both MV-ComBat and MV-CovBat tended to remove unprotected biological signals more aggressively when strong confounding was present, as observed in the A2CPS data (Supplementary Figure S5A,B). In contrast, the univariate framework showed more consistent preservation of unprotected biological variation, likely because it shares less information across features and does not share information across metrics. However, when confounding was weak or absent, the two multivariate frameworks performed better at preserving unprotected biological signals by producing more accurate batch-effect estimates, with MV-CovBat performing slightly better overall (Supplementary Figure S5C). These results highlight the importance of thorough exploratory analyses and batch-effect diagnostics before applying multivariate harmonization, so that covariates to be preserved can be specified appropriately and concerns about unintentionally removing important biological signals can be minimized.

Furthermore, the current framework handles only one batch variable at a time, posing challenges for datasets with hierarchical batch structures, such as nested scanner parameters or software updates. Some studies have combined batch factors pairwise (Beer et al. 2020), but this treats each batch level as independent and ignores potential information sharing (e.g., across scanners from the same vendor). A generalized ComBat method that adjusts batch variables sequentially has shown promise (Horng et al. 2022), yet sequential adjustment may disrupt dependencies among batch variables and amplify noise due to accumulated estimation uncertainty. Future work should aim to incorporate hierarchical structures into harmonization models.

Finally, pooling across metrics may inflate the FDR if non-null signal in some metrics propagates to weaker or null effects in others. Careful selection of metrics for joint harmonization is therefore important. The current MV-ComBat (MCMC) implementation uses a two-stage framework in which fixed effects are first removed and batch effects are then estimated. Although computationally efficient, this design does not fully exploit the Bayesian framework’s ability to jointly estimate fixed and batch effects. Misspecification of the regression model may bias fixed-effect estimates and, in turn, affect batch correction. Future work should explore fully Bayesian models that simultaneously estimate fixed and batch effects and compare their performance with the proposed two-stage approach.

## Supplementary Material

Supplement 1

## Figures and Tables

**Table 1:** Within-metric batch-effect removal performance across experimental conditions (point estimates with 95% confidence intervals) under the model-concordant scenario.

Method	Regular (n = 500)	Regular (n = 150)	Stress Test (n = 100)

MV-ComBat	0.556(0.533, 0.582)	0.600(0.562, 0.640)	0.612(0.572, 0.657)
MV-ComBat (MCMC)	0.559(0.535, 0.585)	0.603(0.564, 0.646)	0.616(0.573, 0.661)
UV-ComBat	0.554(0.533, 0.579)	0.599(0.562, 0.640)	0.604(0.564, 0.647)
unharm	0.999(0.992, 1.000)	0.990(0.965, 1.000)	0.998(0.985, 1.000)

## Data Availability

Data were provided by the A2CPS Consortium funded by the National Institutes of Health (NIH) Common Fund, which is managed by the Office of the Director (OD)/ Office of Strategic Coordination (OSC). Consortium components and their associated funding sources include Clinical Coordinating Center (U24NS112873), Data Integration and Resource Center (U54DA049110), Omics Data Generation Centers (U54DA049116, U54DA049115, U54DA049113), Multi-site Clinical Center 1 (MCC1) (UM1NS112874), and Multi-site Clinical Center 2 (MCC2) (UM1NS118922). The data release used in this analysis is available for download from the NIMH Data Archive (NDA). We integrated UV-ComBat, UV-CovBat, MV-ComBat, and MV-CovBat into a unified R package, MultiComBat, which also includes a suite of commonly used tools for diagnosing batch effects. The design of MultiComBat builds upon the univariate ComBat-family framework as implemented in existing R software ComBatFamily(Chen and Gardner 2024), and extends this framework to the multivariate setting while providing a unified interface and additional diagnostic functionality. The package is available on GitHub at https://github.com/Zheng206/MultiComBat.

## Appendix

### A Derivation for Multivariate EB Batch Adjustment

#### A.1 Framework Specification

In the multivariate ComBat framework, let $Z_{ijv}\in R^{M}$ denote the rescaled residual vector of M metrics for feature v from batch i and subject j, after removing fixed effects. The model assumes the following:

##### 1. Data Likelihood:

The observed data for each feature v in batch i follow a multivariate normal distribution with mean $\gamma_{iv}$ and covariance $\Sigma_{iv}$:

$$Z_{ijv}\overset{\;\text{i.i.d.}\;}{∼}𝒩\left(\gamma_{iv},\Sigma_{iv}\right),\;j=1,\ldots ,n_{i},$$

where $\gamma_{iv}\in R^{M}$ is the batch-specific location effect, and $\Sigma_{iv}\in R^{M\times M}$ is a symmetric positive-definite covariance matrix that captures the batch-specific dispersion across the m metrics for feature v in batch i, shared across subjects j. $n_{i}$ is the number of samples in batch i.

##### 2. Prior for $\gamma_{iv}$:

The mean $\gamma_{iv}$ for each feature v in batch i is modeled with a multivariate normal prior:

$$\gamma_{iv}∼𝒩\left(\gamma_{i},T_{i}\right),$$

where $\gamma_{i}\in R^{M}$ is a batch-level mean and $T_{i}\in R^{M\times M}$ is a covariance matrix that governs how batch means $\gamma_{iv}$ vary *across features* v within batch i.

##### 3. Prior for $\Sigma_{iv}$:

The covariance matrix $\Sigma_{iv}$ for each feature in batch i follows an Inverse-Wishart distribution:

$$\Sigma_{iv}∼IW\left(\Psi_{i},\nu_{i}\right),$$

where $\Psi_{i}$ is the scale matrix and $\nu_{i}$ is the degrees of freedom.

#### A.2 Estimating Hyperparameters

We estimate the prior hyperparameters $\left(\gamma_{i},T_{i},\Psi_{i},\nu_{i}\right)$, and initialize $\gamma_{iv}$ and $\Sigma_{iv}$, using the method of moments.

##### Sample-specific Estimates:

The sample-specific mean ${\overset{ˆ}{\gamma }}_{iv}$ and variance ${\overset{ˆ}{\Sigma }}_{iv}$ estimates are calculated as follows:

(1)
$${\overset{ˆ}{\gamma }}_{iv}=\frac{1}{n_{i}}\sum_{j=1}^{n_{i}}Z_{ijv},\;{\overset{ˆ}{\Sigma }}_{iv}=\frac{1}{n_{i}\;-\;1}\sum_{j=1}^{n_{i}}\left(Z_{ijv}\;-\;{\overset{ˆ}{\gamma }}_{iv}\right){\left(Z_{ijv}\;-\;{\overset{ˆ}{\gamma }}_{iv}\right)}^{⊤},$$

##### Multivariate Normal Prior Hyperparameter Estimates:

The estimates for the hyperparameters of the normal prior are computed as follows:

(2)
$${\overset{ˆ}{\gamma }}_{i}=\frac{1}{p}\sum_{v=1}^{p}{\overset{ˆ}{\gamma }}_{iv},\;{\overset{ˆ}{T}}_{i}=\frac{1}{p\;-\;1}\sum_{v=1}^{p}\left({\overset{ˆ}{\gamma }}_{iv}\;-\;{\overset{ˆ}{\gamma }}_{i}\right){\left({\overset{ˆ}{\gamma }}_{iv}\;-\;{\overset{ˆ}{\gamma }}_{i}\right)}^{⊤},$$

where p is the total number of features.

##### Inverse-Wishart Prior Hyperparameter Estimates:

Moment matching for the Inverse–Wishart prior is a bit more complicated due to the second moments. To simplify, we match only the first moment and the diagonal variances. The estimates for the hyperparameters of the Inverse-Wishart prior are computed as follows:

(3)
$${\overset{ˆ}{V}}_{i}=\frac{1}{p}\sum_{v=1}^{p}{\overset{ˆ}{\Sigma }}_{iv},\;{\overset{ˆ}{s}}_{mm}^{2}=\frac{1}{p\;-\;1}\sum_{v=1}^{p}{\left({\left[{\overset{ˆ}{\Sigma }}_{iv}\right]}_{mm}\;-\;{\left[{\overset{ˆ}{V}}_{i}\right]}_{mm}\right)}^{2},$$

where $[\cdot {]}_{mm}$ denotes the (m,m) element and m=1,…,M.

The sample moments ${\overset{ˆ}{V}}_{i}$ and ${\overset{ˆ}{s}}_{mm}^{2}$ are then used to match the theoretical first moment and diagonal second moment of the Inverse-Wishart distribution. We average across diagonals to obtain a scalar degrees-of-freedom estimate:

$$\bar{V_{mm}^{2}}=\frac{1}{M}\sum_{m=1}^{M}{\left({\left[{\overset{ˆ}{V}}_{i}\right]}_{mm}\right)}^{2},\;\bar{s_{mm}^{2}}=\frac{1}{M}\sum_{m=1}^{M}{\overset{ˆ}{s}}_{mm}^{2}.$$

By solving the moment equations, we obtain

(4)
$${\overset{ˆ}{\nu }}_{i}=M+3+\frac{2\bar{V_{mm}^{2}}}{\bar{s_{mm}^{2}}},\;{\overset{ˆ}{\Psi }}_{i}=\left({\overset{ˆ}{\nu }}_{i}-M-1\right){\overset{ˆ}{V}}_{i}.$$

#### A.3 Obtaining EB Batch Effects

Once the method-of-moments (MoM) hyperparameters are obtained, we apply an empirical Bayes procedure to pool information across features within each batch. This shrinks the feature-specific batch parameters toward the batch-level mean. Specifically, under the priors $\gamma_{iv}∼𝒩\left(\gamma_{i},T_{i}\right)$ and $\Sigma_{iv}∼IW\left(\Psi_{i},\nu_{i}\right)$, the conditional posteriors are available in closed form.

##### Posterior Distribution for $\gamma_{iv}$

$$\pi \left(\gamma_{iv}|Z_{ijg},\Sigma_{iv}\right)\propto exp\left(-\frac{1}{2}\left[\sum_{j=1}^{n_{i}}\;{\left(Z_{ijv}-\gamma_{iv}\right)}^{⊤}\Sigma_{iv}^{-1}\left(Z_{ijv}-\gamma_{iv}\right)+{\left(\gamma_{iv}-\gamma_{i}\right)}^{⊤}T_{i}^{-1}\left(\gamma_{iv}-\gamma_{i}\right)\right]\right)$$

Therefore, the posterior mean of $\gamma_{iv}$ can be expressed as:

$$E\left[\gamma_{iv}|Z_{ijv},\Sigma_{iv}\right]=\Omega_{i}\left(n_{i}\Sigma_{iv}^{-1}{\bar{Z}}_{iv}+T_{i}^{-1}\gamma_{i}\right),$$

where:

$$\Omega_{i}={\left(n_{i}\Sigma_{iv}^{-1}+T_{i}^{-1}\right)}^{-1}$$

and

$${\bar{Z}}_{iv}=\frac{1}{n_{i}}\sum_{j=1}^{n_{i}}Z_{ijv}.$$

Given ${\overset{ˆ}{\gamma }}_{iv}$, ${\overset{ˆ}{\gamma }}_{i}$, ${\overset{ˆ}{T}}_{i}$, and ${\overset{ˆ}{\Sigma }}_{iv}$ as defined above, the posterior mean can be estimated as:

(5)
γiv*=E^γiv∣Zijv,Σiv=niΣiv*-1+Tˆi-1-1niΣiv*-1Z¯iv+Tˆi-1γˆi,

##### Posterior Distribution for $\Sigma_{iv}$

$$\pi \left(\Sigma_{iv}|Z_{ijv},\gamma_{iv}\right)\propto {\left|\Sigma_{iv}\right|}^{-\frac{n_{i}+\nu_{i}+M+1}{2}}exp\left(-\frac{1}{2}tr\left(\left(\sum_{j=1}^{n_{i}}\;\left(Z_{ijv}-\gamma_{iv}\right){\left(Z_{ijv}-\gamma_{iv}\right)}^{⊤}+\Psi_{i}\right)\Sigma_{iv}^{-1}\right)\right).$$

The posterior mean for $\Sigma_{iv}$ is then:

$$E\left[\Sigma_{iv}|Z_{ijv},\gamma_{iv}\right]=\frac{\sum_{j=1}^{n_{i}}\;\left(Z_{ijv}\;-\;\gamma_{iv}\right){\left(Z_{ijv}\;-\;\gamma_{iv}\right)}^{⊤}\;+\;\Psi_{i}}{n_{i}\;+\;\nu_{i}\;-\;M\;-\;1}.$$

Given ${\overset{ˆ}{\nu }}_{i}$ and ${\overset{ˆ}{\Psi }}_{i}$ as defined above, the posterior mean can be estimated as:

(6)
Σiv*=E^Σiv∣Zijv,γiv=∑j=1niZijv-γiv*Zijv-γiv*⊤+Ψˆini+νˆi-M-1.

#### B Derivation of G Space via the Rayleigh-Ritz Theorem

We consider

$$\underset{G\in R^{n\times r_{s}}}{max}\;Tr\left(G^{⊤}SG\right)\;\;\text{subject}\;\text{to}\;\;G^{⊤}G=I_{r_{s}},$$

where $S=\sum_{m=1}^{M}F^{\left(m\right)}F^{(m)⊤}$ is symmetric and positive semidefinite.

Let $S=U\Lambda U^{⊤}$ be its eigendecomposition, with $\Lambda =diag\left(\lambda_{1},\ldots ,\lambda_{n}\right)$ and $\lambda_{1}\geq \cdots \geq \lambda_{n}\geq 0$. Substituting gives

$$Tr\left(G^{⊤}SG\right)=Tr\left(G^{⊤}U\Lambda U^{⊤}G\right)=Tr\left(H^{⊤}\Lambda H\right),$$

where $H=U^{⊤}G$ and $H^{⊤}H=I_{r_{s}}$.

By the Rayleigh-Ritz theorem, this quantity is maximized when the columns of H select the top $r_{s}$ eigenvectors of Λ, yielding

$$\underset{G^{⊤}G=I_{r_{s}}}{max}\;Tr\left(G^{⊤}SG\right)=\lambda_{1}+\cdots +\lambda_{r_{s}},\;G^{*}=U_{\left[:,1:r_{s}\right]}.$$

Hence, the maximizer $G^{*}$ consists of the top $r_{s}$ eigenvectors of

$$S=\sum_{m=1}^{M}F^{\left(m\right)}F^{(m)⊤}.$$
