# Supplementary material for: MV-ComBat and MV-CovBat: Multivariate Frameworks for Joint Harmonization of Multi-Metric Neuroimaging Data
Source: bioRxiv. 2026 Feb 9:2026.02.05.704069. Preprint. [Version 1] doi: 10.64898/2026.02.05.704069 (PMC12919036; doi:10.64898/2026.02.05.704069)
Supplement: Supplement 1 [file NIHPP2026.02.05.704069v1-supplement-1.pdf]

bioRxiv preprint doi: https://doi.org/10.64898/2026.02.05.704069; this version posted February 9, 2026. The copyright holder for this preprint (which was not certified by peer review) is the author/funder, who has granted bioRxiv a license to display the preprint in perpetuity. It is made available under aCC-BY 4.0 International license.

Supplementary Materials

S1 Bayesian Model and MCMC Implementation

The multivariate Bayesian model was fit using Markov Chain Monte Carlo (MCMC) sampling implemented in Stan. We ran three independent chains in parallel, with an adaptive Hamiltonian Monte Carlo sampler using a target acceptance rate of 0.98. Batch-level intercepts were assigned standard normal priors. Feature- and batch-specific mean vectors were modeled with normal priors centered on the corresponding batch intercepts. Standard deviation parameters were assigned half-Student-t priors with degrees of freedom drawn from a Gamma$(2, 0.1)$ distribution and scale parameters drawn from a Cauchy$(0, 2.5)$ distribution. Correlation structures were modeled using LKJ priors with shape parameter 1. A complete specification of prior distributions is provided in Supplementary Table S3. Convergence was assessed using standard diagnostics provided by Stan, including the potential scale reduction factor ($\hat{R}$) and effective sample size.

S2 Supplementary Figure

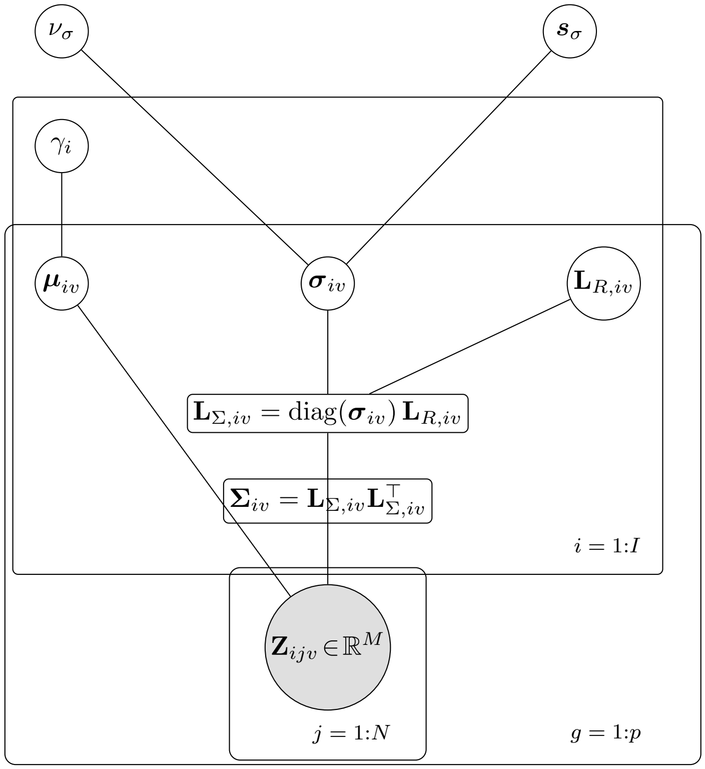

$$
\begin{aligned}
\nu_\sigma &\sim \mathrm{Gamma}(2, 0.1)\\
\boldsymbol{s}_\sigma &\sim \mathrm{Cauchy}(0, 2.5)\\
\gamma_i &\sim \mathcal{N}(0, 1)\\
\boldsymbol{\mu}_{iv} &\sim \mathcal{N}(\gamma_i \mathbf{1},\ 2^2\mathbf{I})\\
\boldsymbol{\sigma}_{iv} &\sim t^+_{\nu_\sigma}(0, \boldsymbol{s}_\sigma)\\
\mathbf{L}_{R,iv} &\sim \mathrm{LKJ\_corr\_chol}(1)\\
\mathbf{Z}_{ijv} &\sim \mathcal{N}(\boldsymbol{\mu}_{iv},\ \boldsymbol{\Sigma}_{iv})
\end{aligned}
$$

Figure S1: Plate diagram corresponding to the Stan model. Shaded = observed, white = latent, rounded rectangle = deterministic transform. Bold symbols are M-vectors.

bioRxiv preprint doi: https://doi.org/10.64898/2026.02.05.704069; this version posted February 9, 2026. The copyright holder for this preprint (which was not certified by peer review) is the author/funder, who has granted bioRxiv a license to display the preprint in perpetuity. It is made available under aCC-BY 4.0 International license.

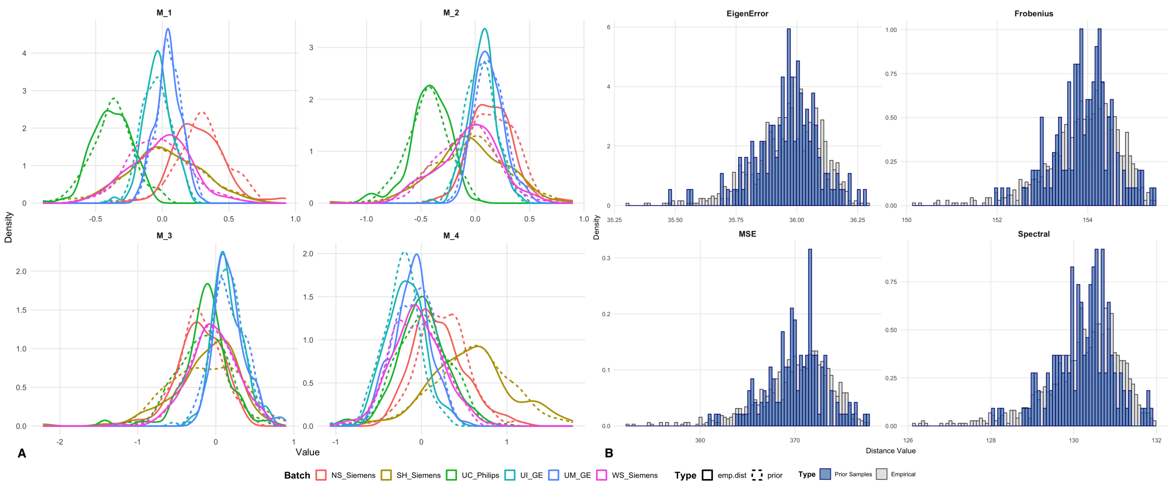

Figure S2: EB Assumption Check. (A) Comparison of the empirical distribution of estimated additive batch effects with the corresponding gamma prior. (B) Prior predictive check for the IW prior used for the multiplicative batch effects.

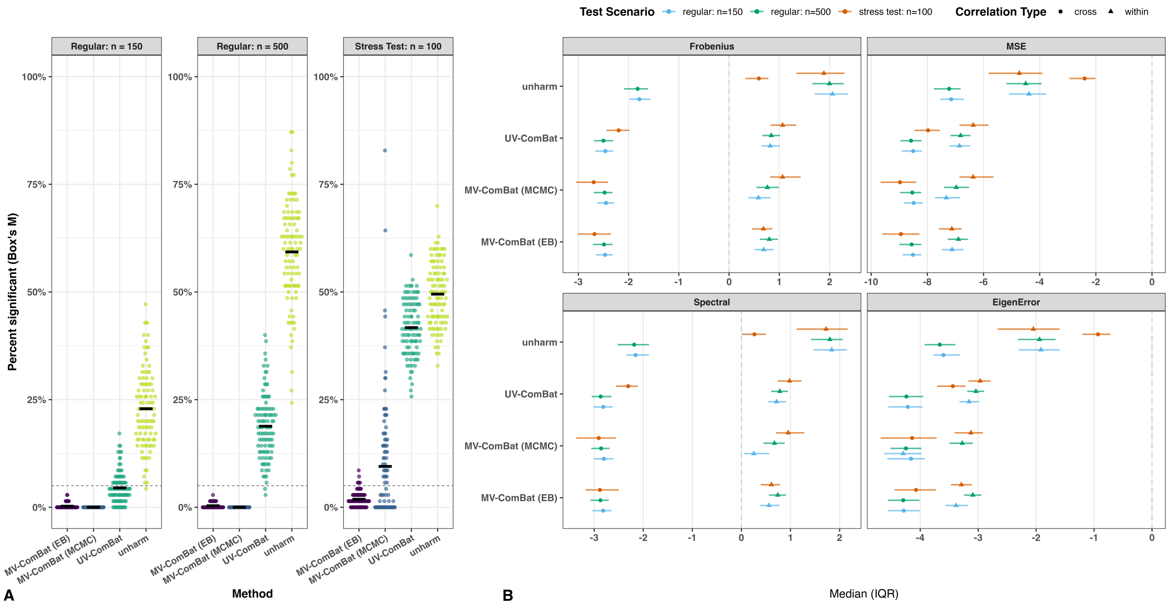

Figure S3: Batch Detection in Covariance and Correlation Recovery (Model-Misspecified; c.f., Figure 4). (A) Box's M test across experimental conditions. Both MV-ComBat variants outperformed UV-ComBat, with the MCMC variant showing greater gains under mild batch effects, but becoming less stable under stress. (B) Correlation recovery. The MCMC approach performed similarly to the EB approach.

bioRxiv preprint doi: https://doi.org/10.64898/2026.02.05.704069; this version posted February 9, 2026. The copyright holder for this preprint (which was not certified by peer review) is the author/funder, who has granted bioRxiv a license to display the preprint in perpetuity. It is made available under aCC-BY 4.0 International license.

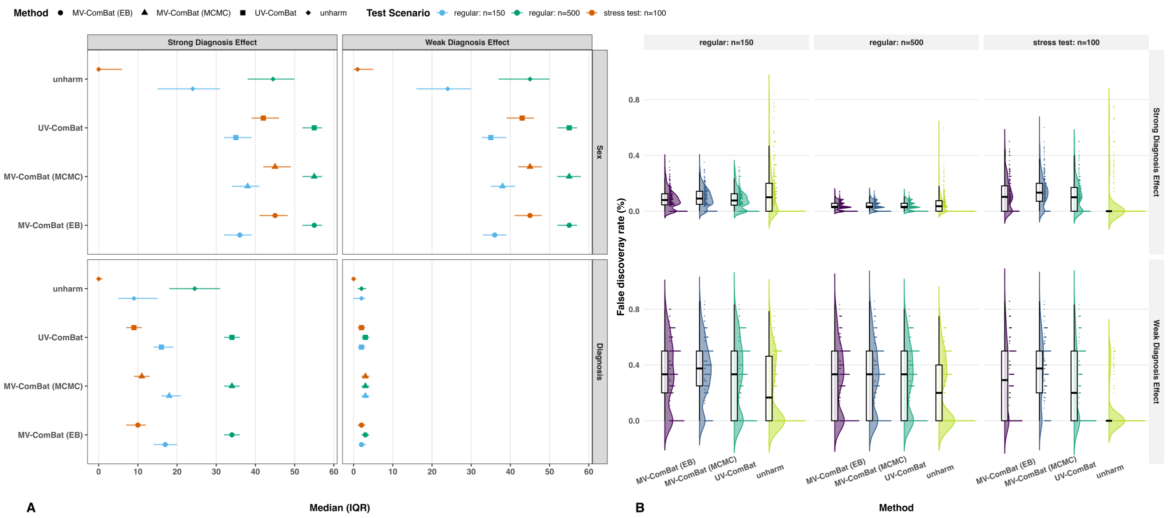

Figure S4: Fixed-effects Detection and Corresponding FDRs (Model-Misspecified; c.f., Figure 6). (A) Preservation of biological signals. Counts of features with significant sex and diagnosis effects across experimental conditions. MV-ComBat (MCMC) performed similarly to MV-ComBat (EB). (B) False discovery rate (FDR) based on known biomarkers. Both MV-ComBat variants performed comparably to UV-ComBat.

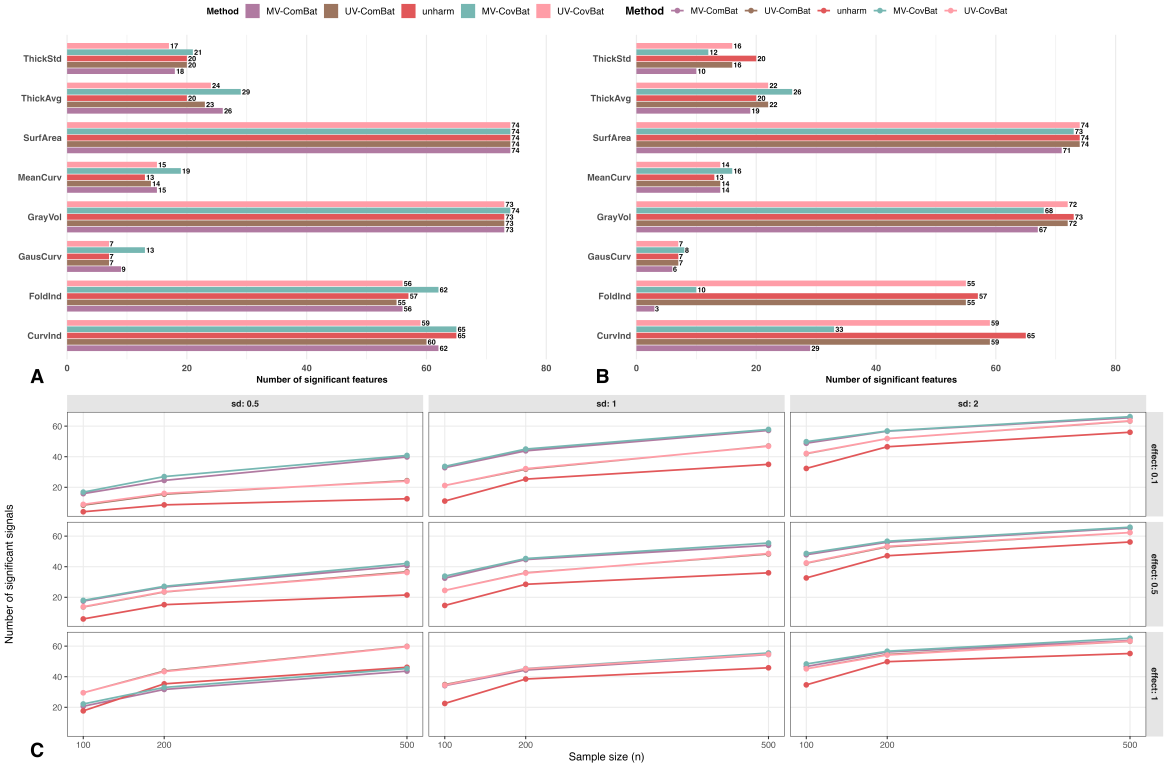

Figure S5: Biological signal preservation. (A) Preservation of sex effects in the A2CPS data when sex is included as a covariate, shown as the number of features with significant sex effects. (B) Preservation of sex effects in the A2CPS data when sex is not included as a covariate, shown as the number of features with significant sex effects. (C) Preservation of an omitted biological signal in simulations varying by effect size, signal standard deviation, and sample size.

bioRxiv preprint doi: https://doi.org/10.64898/2026.02.05.704069; this version posted February 9, 2026. The copyright holder for this preprint (which was not certified by peer review) is the author/funder, who has granted bioRxiv a license to display the preprint in perpetuity. It is made available under aCC-BY 4.0 International license.

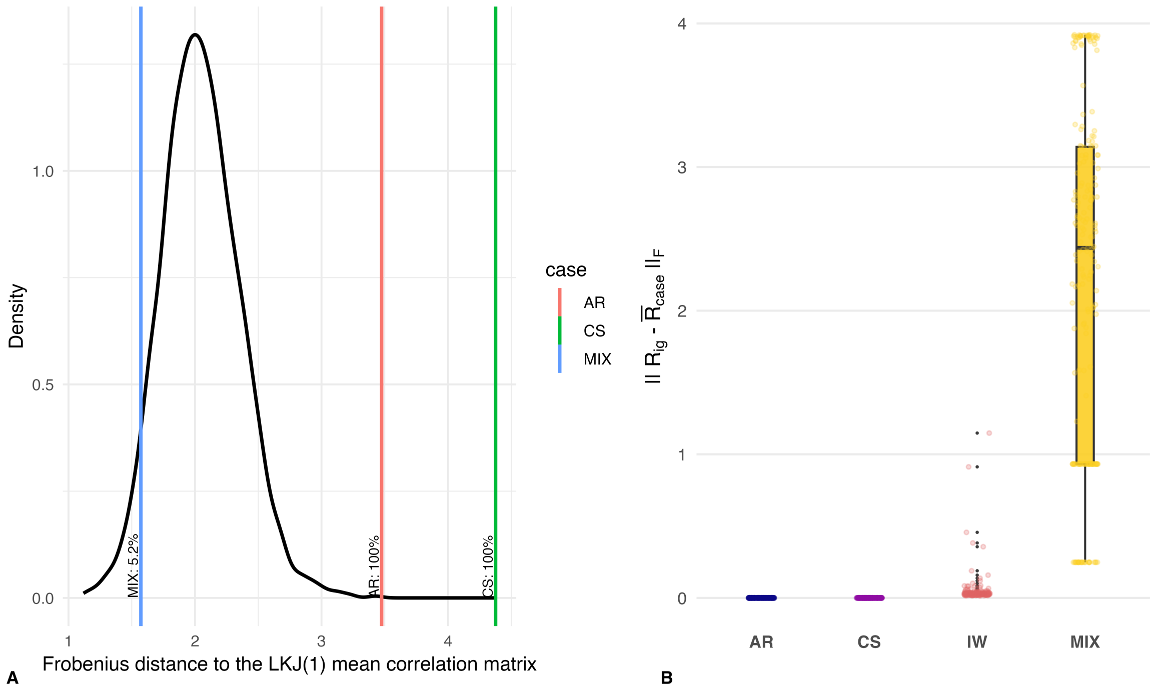

Figure S6: Prior-data alignment and feature-wise heterogeneity across covariance regimes. (A) Distribution of Frobenius distances between correlation matrices drawn from an LKJ(1) prior and the LKJ(1) mean correlation matrix (black density). Vertical lines indicate the Frobenius distance between the case-averaged simulated correlation matrix and the LKJ(1) mean for each data-generating regime (AR, CS, MIX). Correlations generated under the AR and CS regimes lie far outside the typical support of the LKJ(1) prior, indicating substantial prior–data mismatch. In contrast, the MIX regime exhibits a markedly smaller distance, which arises from averaging across heterogeneous, feature-specific covariance regimes rather than from genuine alignment with the LKJ prior. (B) Feature-wise deviations of individual correlation matrices from the case-average correlation. While the AR and CS regimes show minimal within-case variability, the MIX regime exhibits extreme feature-level heterogeneity, with large dispersion across features. Taken together, these patterns help explain why exchangeable priors such as the LKJ can obscure feature-specific structure and degrade performance under heterogeneous covariance regimes, particularly in data-limited or high-noise settings.

S3 Supplementary Table

Table S1: Three experimental conditions and parameter settings used across scenarios.

**Experiment**	**n**	β_{gm}^{Age}	β_{gm}^{Sex}	β_{gm}^{Diag}	$\boldsymbol{\gamma}_i$	$\mathbf{T}_i$
Stress	100	$\mathcal{N}(2,\ 0.3^2)$	$\mathcal{N}(1.8,\ 0.3^2)$	$\mathcal{N}(\mu_m,\ 0.1^2)$	$\mathcal{N}(\mathbf{0},\ 30^2\mathbf{I})$	$\mathbf{DRD} + 45\,i\,\mathbf{I}_M$
Regular A	150	$\mathcal{N}(0.7,\ 0.2^2)$	$\mathcal{N}(0.5,\ 0.15^2)$	$\mathcal{N}(\mu_m,\ 0.1^2)$	$\mathcal{N}(\mathbf{0},\ 3^2\mathbf{I})$	$\mathbf{DRD} + 0.3\,i\,\mathbf{I}_M$
Regular B	500	$\mathcal{N}(0.7,\ 0.2^2)$	$\mathcal{N}(0.5,\ 0.15^2)$	$\mathcal{N}(\mu_m,\ 0.1^2)$	$\mathcal{N}(\mathbf{0},\ 3^2\mathbf{I})$	$\mathbf{DRD} + 0.3\,i\,\mathbf{I}_M$
**Additional parameters**	$\nu_i = M + 2$, $\boldsymbol{\Psi}_i = \mathbf{DRD} + \sigma\,i\,\mathbf{I}_M$, $\sigma = 45$ under stress test, otherwise $\sigma = 0.3$.					

Note. $\mathbf{D} = \text{diag}(d_1, \ldots, d_M)$ is a diagonal scaling matrix with $d_m \sim \mathcal{N}(1, 0.8^2)$. $\mathbf{R}$ denotes the base correlation matrix, chosen as AR(1) with correlation parameter $\rho = 0.8$ and marginal variance $\sigma^2 = 1$. The term $\sigma\,i\,\mathbf{I}_M$ increases the overall covariance magnitude proportionally to the batch index i.

bioRxiv preprint doi: https://doi.org/10.64898/2026.02.05.704069; this version posted February 9, 2026. The copyright holder for this preprint (which was not certified by peer review) is the author/funder, who has granted bioRxiv a license to display the preprint in perpetuity. It is made available under aCC-BY 4.0 International license.

Table S2: Covariance generators used in the model-misspecified mixture.

Component	Correlation $\mathbf{R}$	Scales $\boldsymbol{\sigma}$	Covariance $\boldsymbol{\Sigma}$
IW	—	—	$\boldsymbol{\Sigma} \sim \mathrm{IW}(\nu_i, \boldsymbol{\Psi}_i)$
LKJ	$\mathbf{R} \sim \mathrm{LKJ}(\eta = 2)$	$\sigma_m \overset{\text{i.i.d.}}{\sim} \text{Half-}t_4(0,1)$	$\mathbf{DRD}$
FA	—	$\psi_m \overset{\text{i.i.d.}}{\sim} \mathrm{Unif}(0.2, 1.2)$	$\boldsymbol{\Lambda}\boldsymbol{\Lambda}^\top + \boldsymbol{\Psi}_{\mathrm{FA}}$
AR	$R_{mn} = \rho^{\lvert m-n \rvert},\ \rho = 0.6$	$\sigma_m \overset{\text{i.i.d.}}{\sim} \text{Half-}t_4(0,1)$	$\mathbf{DRD}$
CS	$R_{mn} = \rho\ ,\ R_{mm} = 1,\ \rho = 0.3$	$\sigma_m \overset{\text{i.i.d.}}{\sim} \text{Half-}t_4(0,1)$	$\mathbf{DRD}$

$\mathbf{D} = \mathrm{diag}(\boldsymbol{\sigma})$. For the FA component, $\boldsymbol{\Lambda}$ is an $M \times r$ loading matrix with entries $\Lambda_{mr} \sim \mathcal{N}(0, 0.7^2)$ and rank $r \leq \min(2, M)$; $\boldsymbol{\Psi}_{\mathrm{FA}} = \mathrm{diag}(\psi_1, \ldots, \psi_M)$ represents feature-specific uniquenesses.

**Method**	**Regular (n = 500)**	**Regular (n = 150)**	**Stress Test (n = 100)**
MV-ComBat	0.555(0.533, 0.580)	0.596(0.559, 0.635)	0.604(0.567, 0.646)
MV-ComBat (MCMC)	0.560(0.535, 0.586)	0.605(0.567, 0.646)	0.601(0.566, 0.645)
UV-ComBat	0.555(0.532, 0.579)	0.600(0.565, 0.643)	0.604(0.563, 0.644)
unharm	0.999(0.992, 1.000)	0.990(0.965, 1.000)	1.000(0.990, 1.000)

Table S3: Within-metric batch-effect removal performance across experimental conditions (point estimates with 95% confidence intervals) under the model-misspecified scenario.

**Component**	**Parameter**	**Description**	**Prior / Setting**
Hyperparameters	ν_σ	Degrees of freedom for Half-t prior on $\boldsymbol{\sigma}_{iv}$	$\mathrm{Gamma}(2, 0.1)$
	s_σ	Scale parameter for Half-t prior	$\mathrm{Cauchy}(0, 2.5)$
Batch-level effects	γ_i	Batch-level intercept for batch i	$\mathcal{N}(0, 1)$
Mean structure	$\boldsymbol{\mu}_{iv}$	Feature- and batch-specific mean vector ($\in \mathbb{R}^M$)	$\mathcal{N}\big(\gamma_i \mathbf{1},\ 2^2\mathbf{I}\big)$
Scale parameters	$\boldsymbol{\sigma}_{iv}$	Feature- and batch-specific standard deviations ($\in \mathbb{R}^M_+$)	$t^+_{\nu_\sigma}(0, s_\sigma)$
Correlation structure	$\mathbf{L}_{R,iv}$	Cholesky factor of correlation matrix	LKJ_corr_chol(1)
Covariance construction	$\boldsymbol{\Sigma}_{iv}$	Feature- and batch-specific covariance matrix	$\boldsymbol{\Sigma}_{iv} = \mathbf{L}_{\Sigma,iv}\mathbf{L}_{\Sigma,iv}^\top$
	$\mathbf{L}_{\Sigma,iv}$	Cholesky factor of covariance matrix	$\mathbf{L}_{\Sigma,iv} = \mathrm{diag}(\boldsymbol{\sigma}_{iv})\mathbf{L}_{R,iv}$
Likelihood	$\mathbf{Z}_{ijv}$	Observed measurement vector for subject j ($\in \mathbb{R}^M$)	$\mathcal{N}(\boldsymbol{\mu}_{iv}, \boldsymbol{\Sigma}_{iv})$
MCMC sampler	—	Sampling algorithm	Hamiltonian Monte Carlo (Stan; NUTS)
Number of chains	—	Independent MCMC chains	3
Parallel chains	—	Chains run in parallel	3
Adaptation	—	Target acceptance probability	0.98
Diagnostics	—	Convergence assessment	$\hat{R}$ and effective sample size (ESS)
Posterior summaries	—	Reported estimates	Posterior means

Table S4: Prior distributions and MCMC configuration for the multivariate Bayesian model.
